# Immune-based subgroups uncover diverse tumor immunogenicity and implications for prognosis and precision therapy in acute myeloid leukemia

**DOI:** 10.3389/fimmu.2024.1451486

**Published:** 2024-11-08

**Authors:** Tingting Chen, Yue Zhang, Danyang Zhang, Hebing Zhou

**Affiliations:** Department of Hematology, Beijing Luhe Hospital Affiliated to Capital Medical University, Beijing, China

**Keywords:** acute myeloid leukemia, tumor microenvironment, immune subgroups, prognosis, drug sensitivity

## Abstract

**Background:**

Although a considerable proportion of acute myeloid leukemia (AML) patients achieve remission through chemotherapy, relapse remains a recurring and significant event leading to treatment failure. This study aims to investigate the immune landscape in AML and its potential implications for prognosis and chemo-/immune-therapy.

**Methods:**

Integrated analyses based on multiple sequencing datasets of AML were performed. Various algorithms estimated immune infiltration in AML samples. A subgroup prediction model was developed, and comprehensive bioinformatics and machine learning algorithms were applied to compare immune-based subgroups in relation to clinical features, mutational landscapes, immune characterizations, drug sensitivities, and cellular hierarchies at the single-cell level.

**Results:**

Two immune-based AML subgroups, G1 and G2, were identified. G1 demonstrated higher immune infiltration, a more monocytic phenotype, increased proportions of monocytes/macrophages, and higher FLT3, DNMT3A, and NPM1 mutation frequencies. It was associated with a poorer prognosis, lower proportions of various immune cell types and a lower T cell infiltration score (TIS). AML T-cell-based immunotherapy target antigens, including CLEC12A, Folate receptor β, IL1RAP and TIM3, showed higher expression levels in G1, while CD117, CD244, CD96, WT and TERT exhibited higher expression levels in G2. G1 samples demonstrated higher sensitivity to elesclomol and panobinostat but increased resistance to venetoclax compared to G2 samples. Moreover, we observed a positive correlation between sample immune infiltration and sample resistance to elesclomol and panobinostat, whereas a negative correlation was found with venetoclax resistance.

**Conclusion:**

Our study enriches the current AML risk stratification and provides guidance for precision medicine in AML.

## Introduction

Acute myeloid leukemia (AML) is a prevalent hematological cancer characterized by the clonal expansion of undifferentiated myeloid progenitor cells (1). It exhibits significant heterogeneity in clinical courses and responses to therapy (2).

Although the standard treatment approach for AML has demonstrated success in achieving complete remission, a notable percentage of patients either struggle to endure intensive initial treatment due to frailty or encounter relapse with highly resistant disease (3, 4). Consequently, the chances of long-term survival beyond a five-year period stand at a mere 31.7% (5), while the likelihood of a complete recovery for individuals above 60 years of age varies between 5% and 15% (6).

Therefore, it holds immense clinical significance to conduct further investigation into the genomic characteristics and therapeutic targets associated with AML.

It is widely acknowledged that immune cells within the tumor microenvironment (TME) play a pivotal role in recognizing and eliminating cancer cells through various immune mechanisms, a phenomenon referred to as immunosurveillance (7). The involvement of immune cells and stromal cells as major components in leukemogenesis and disease progression has been well established (8–10). Despite the growing body of research highlighting the significance of immunotherapy for AML (11–14), there is still limited clarity and a lack of comprehensive understanding regarding the immune landscape and molecular characteristics of the AML TME.

In this study, we aimed to investigate the immune landscape within the AML TME using bulk RNA sequencing (RNA-seq) data, along with somatic mutation and single-cell RNA data. We identified key AML immune-related genes (IRGs), categorized AML samples into two distinct immune subgroups, developed a subgroup prediction model and compared these subgroups based on clinical features, immune characteristics, mutation patterns, drug responses, and cellular hierarchies. Our comprehensive analysis yielded valuable insights into the immune landscape of AML and highlighted its potential clinical significance for prognostic stratification and personalized medicine approaches in the treatment of AML patients.

## Materials and methods

### Data acquisition and preprocessing

The latest summary of 2,483 immune-related genes (IRGs) was downloaded from the Immunology Database and Analysis Portal (ImmPort, https://www.immport.org/home (15), last accessed on May 5th, 2023) for further investigation.

We conducted a comprehensive search for publicly available transcriptome data of *de novo* AML samples and normal whole blood samples with clinical annotations. Our study included three RNA-Seq cohorts:

TCGA cohort: We retrieved the expression profiles and clinical information of 173 TCGA-LAML samples (16, 17) from the UCSC Xena database (http://xena.ucsc.edu/) (19), specifically from the “cohort: TCGA Acute Myeloid Leukemia (LAML)”. The inclusion criteria for this cohort stipulated that AML samples must have both gene expression and phenotype data.

GTEx (18) cohort: A total of 337 normal whole blood samples were sourced from the “cohort: GTEX” in the UCSC Xena database. Inclusion criteria for the GTEx cohort included: 1) normal whole blood samples; 2) availability of both gene expression RSEM expected counts data and phenotype data within the same cohort.

Beat AML cohort: Gene expression data and clinical information for the Beat AML cohort samples were obtained from the online browser (http://www.vizome.org/) provided by Jeffrey W. Tyner et al., as well as their original studies (20, 21). Inclusion criteria for the Beat AML cohort included: 1) newly diagnosed AML and 2) specimen type of either bone marrow or peripheral blood. A total of 255 samples were included in this study.

Additionally, we incorporated a single-cell RNA sequencing (scRNA-Seq) dataset, GSE116256 (22, 23), which included sixteen *de novo* AML samples. The read count matrices, sample clinical data, cell type annotation information, and cell type signature genes for the scRNA-Seq dataset were obtained from the GEO database and previous studies (22, 23).

The somatic mutation data of the TCGA cohort, identified using MuTect2, were obtained in the mutation annotation format (MAF) files and analyzed using the R package “maftools”. For our study, we included somatic mutation data from a total of 166 samples from the TCGA cohort that also had RNA expression profiles.

The somatic mutation data and the ex vivo drug sensitivity data for the Beat AML cohort were obtained from the original studies (20, 21).

### Immune infiltration analysis

We employed two algorithms, namely ESTIMATE (24) and xCell (25), to quantify the immune and stromal scores in heterogeneous AML samples using their transcriptome data. These analyses were conducted using the R packages “estimate” and “xCell”. Additionally, we utilized the CIBERSORT method (26) to estimate the abundances of infiltrating immune cells in the AML samples.

To calculate the T cell infiltration score (TIS), we adopted the definition proposed by Rooney MS, et al. (27). In accordance with their definition, the TIS is determined by summing the absolute abundances of the following immune cell types: CD8+ T cell, CD4+ naïve T cell, CD4+ memory T cell, T follicular helper cell, regulatory T cell, and γδ T cell.

### Identification of AML subgroups with AML-specific IRGs

The AML-specific IRGs were obtained using R package “DESeq2”, identified as differentially expressed IRGs between AMLs from the TCGA cohort and normal whole blood (WB) samples from the GTEx cohort, satisfying the filtering criteria of padj < 0.01 and |log2(Foldchange)| > 1.

After filtering the AML-specific IRGs, retaining only the genes with counts greater than 0 in at least 20% (35/173) of the AML samples in the TCGA cohort, an unsupervised analysis was conducted in the TCGA cohort. This analysis was based on the expression profiles of the filtered genes, using the “ConsensusClusterPlus” R package (28). Consensus clustering, known for its robustness and insensitivity to random starts compared to traditional k-means and hierarchical clustering algorithms, has been widely used to identify biologically meaningful clusters (28). We explored a range of cluster numbers from 2 to 10 and selected the optimal number that yielded the most stable consensus matrices and unambiguous cluster assignments across multiple permutations of clustering runs (29, 30). The final clusters corresponded to the intrinsic subgroups of AML that were identified.

### The identification of key-AML-IRGs

We employed Weighted Gene Co-expression Network Analysis (WGCNA) using the R package “WGCNA” to construct a scale-free co-expression network (31). This analysis allowed us to identify “AML-immune-specific” modules that exhibited a strong correlation with immune scores in AML.

The associations between individual genes and the trait of interest (namely immune scores here) were determined using gene significance (GS), which quantifies the correlation between genes and clinical traits. Additionally, module membership (MM) was defined to measure the relevance between module eigengenes and gene expression profiles. Ultimately, genes exhibiting high GS for immune scores and high MM in AML-immune-specific modules were identified as “key-AML-IRGs” in AML patients.

### The establishment of subgroup prediction model

We utilized AutoGluon (v0.2.0), a Python library available at https://github.com/awslabs/autogluon (32), to construct a subgroup prediction model based on the expression profiles of the key-AML-IRGs. AutoGluon is a powerful tool for automated machine learning (AutoML) that focuses on automated stack ensembling, deep learning, and real-world applications spanning image, text, and tabular data. It enables easy-to-use and easy-to-extend AutoML capabilities (source: https://auto.gluon.ai/dev/index.html). Within the given resources, AutoGluon trains a diverse range of models to leverage their collective predictions, making it an effective strategy.

In this study, the models employed within the AutoGluon framework include CatBoost, ExtraTreesEntr, ExtraTreesGini, KNeighborsDist, KNeighborsUnif, LightGBM, LightGBMLarge, LightGBMXT, NeuralNetFastAI, NeuratNetTorch, RandomForestEntr, RandomForestGini, WeightedEnsemble, and XGBoost models that aggregate full-layer results. For model training, the TCGA cohort served as the training cohort, with the samples randomly split into a training group and a test group using an 8:2 ratio. The model with the best performance was selected. Subsequently, we validated the prognostic value of the established subgroup prediction model by applying it to an independent cohort, namely the Beat AML cohort.

### Single-Cell Identification of Subpopulations with bulk Sample phenotype correlation analysis

The single-cell raw matrix data of 16 *de novo* AML samples from GSE116256 were downloaded and imported using the “Seurat” R package. Filtering parameters were set as min.cells = 3, percent.mt < 5, and nFeature_RNA < 3000 (**Supplementary Figure S1**). This filtering process resulted in a final dataset containing 15,058 cells and 18,515 genes.

Gene signatures for dataset GSE116256 were obtained from the study by Galen et al. (23). To map the gene signatures of various differentiation states to bulk samples from the TCGA and Beat AML cohorts, we employed methods similar to those used in previous studies (33). For each bulk sample, genes with a total count below 35 across all samples were filtered out. The expression data were then normalized using z-scores. We selected the top 100 genes with the greatest deviations from the mean expression to serve as background genes. The mean expression of the signature genes for each cell type was calculated, and signature scores for each bulk sample were derived by subtracting the average expression of the background genes from that of the signature genes.

To identify biologically and clinically relevant cell subsets in single-cell RNA sequencing, we employed Scissor algorithm (34). Scissor utilizes bulk data and phenotype information to accurately and specifically identify cell subsets. The method integrates phenotype-associated bulk expression data and single-cell data by assessing the similarity between each single cell and each bulk sample. It then employs a regression model optimized on the correlation matrix with the sample phenotype to identify relevant subpopulations (34).

In our study, we performed Scissor analysis using the normalized expression profiles of 16 *de novo* AML samples from GSE116256, along with the expression and subgrouping information of the TCGA cohort. The subgrouping data of the TCGA cohort was utilized as a binary variant, and Scissor analysis was applied to the scRNA-Seq data of the 16 AML samples to identify cells characterized by G1 or G2 subgroups. The results of the Scissor analysis assigned labels of “BC” to background cells, “Scissor+” to cells characterized by strong G1 signatures, and “Scissor-” to cells characterized by strong G2 signatures.

### Additional bioinformatic and statistical analyses

ANOVA was employed to determine if there were significant differences among more than two groups. This was followed by Tukey’s HSD test to further compare the significance between each pair of groups. The Kaplan-Meier method was employed to generate survival curves, and the log-rank test was conducted to assess survival differences. These analyses were carried out using the R packages “survival” and “survminer”. Additionally, multivariate Cox regression analysis was performed to evaluate the significance of each variable for survival, and the results were visualized using the R package “survmine”.

PCA was utilized to visualize the dissimilarity between samples belonging to the G1 and G2 subgroups.

The Robust Rank Aggregation (RRA) algorithm (35), implemented using the R package “RobustRankAggreg”, was employed to identify the most statistically relevant genes from the differentially expressed gene (DEG) list of the G1 and G2 subgroups in the TCGA and Beat AML cohorts. The RRA algorithm compares the actual rankings of genes with the expected behavior of uncorrelated rankings. It re-ranks the genes and assigns significance scores based on their robustness (35). The genes identified through this process were considered as robust DEGs for further analysis (36).

Gene Set Enrichment Analysis (GSEA) (37) was performed using the transcriptome data in conjunction with the hallmark gene sets obtained from the Molecular Signatures Database (MSigDB) (38). GSEA allows for the identification of biological pathways and processes that are significantly enriched within the dataset, providing insights into the underlying functional implications of gene expression patterns (37).

The CytoTRACE algorithm was employed to assess the differentiation status of Scissor-labeled cells using scRNA-Seq data GSE116256. CytoTRACE is a robust computational framework for resolving single-cell differentiation hierarchies. It captures, smooths, and calculates the expression levels of genes highly correlated with single-cell gene counts in scRNA-Seq data. After the calculation, each single cell is assigned a CytoTRACE score representing its differentiation state within the dataset. CytoTRACE scores range from 0 to 1, with higher scores indicating lower differentiation levels and vice versa (39, 40).

### Contact for resource and code sharing

All public data used in this study, along with key software and algorithms, are listed in **Table 1**. Further information and requests for resources and code should be directed to the Lead Contact, Hebing Zhou (bjlhyyxyk@ccmu.edu.cn).

**Table 1:** Key resources table.

RESOURCE		SOURCE	IDENTIFIER
Datasets			
Immune-related genes (IRGs)		ImmPort	https://www.immport.org/home
TCGA-LAML	RNA sequencing and clinical data	Xena	https://xenabrowser.net/datapages/
	Somatic mutation data	R version 4.3.0	https://www.bioconductor.org/packages/release/bioc/html/maftools.html
GTEx	RNA sequencing and clinical data	Xena	https://xenabrowser.net/datapages/
Beat AML	RNA sequencing and clinical data	Vizome	http://www.vizome.org/
	Somatic mutation data	Bottomly, D., et al.	https://www.ncbi.nlm.nih.gov/pmc/articles/PMC9378589/
	Ex Vivo Drug Sensitivity Data	Bottomly, D., et al.	https://www.ncbi.nlm.nih.gov/pmc/articles/PMC9378589/
GSE116256	scRNA sequencing and clinical data	GEO	https://www.ncbi.nlm.nih.gov/geo/query/acc.cgi?acc=GSE116256
Software and Algorithms			
AutoGluon		Github	https://github.com/awslabs/autogluon
Python 3.8		Python Software Foundation (PSF)	https://www.python.org/
R version 4.3.0		R Core Team	https://www.r-project.org
R package CIBERSORT		Stanford	http://cibersort.stanford.edu/
R package ConsensusClusterPlus		Bioconductor	https://bioconductor.org/packages/release/bioc/html/ConsensusClusterPlus.html
R package CytoTRACE		Stanford	https://cytotrace.stanford.edu/
R package DESeq2		Bioconductor	https://bioconductor.org/packages/release/bioc/html/DESeq2.html
R package ESTIMATE		SOURCEFORGE	https://sourceforge.net/projects/estimateproject/
R package maftools		Bioconductor	https://bioconductor.org/packages/release/bioc/html/maftools.html
R package RobustRankAggre		CRAN	https://cran.r-project.org/web/packages/RobustRankAggreg/index.html
R package Scissor		Github	https://github.com/sunduanchen/Scissor
R package Seurat		CRAN	https://cran.r-project.org/web/packages/Seurat/index.html
R package survival		CRAN	https://cran.r-project.org/web/packages/survival/index.html
R package survminer		CRAN	https://cran.r-project.org/web/packages/survminer/index.html
R package WGCNA		CRAN	https://cran.r-project.org/web/packages/WGCNA/index.html
R package xCell		Github	https://github.com/dviraran/xCell

## Results

### Immune conditions are associated with clinical characteristics in AML samples

We evaluated the infiltrating levels of immune and stromal cells involved in AML TME by analyzing the transcriptome data of two independent cohorts, the TCGA cohort (n = 173) and the Beat AML cohort (n = 255) (please refer to **Supplementary Tables S1**, **S2** for the baseline information of the samples). Two distinct algorithms, namely ESTIMATE and xCell, were employed to enhance the reliability of our findings. Based on **Supplementary Figures S2A–H**, it is evident that in AML samples, the immune scores (red boxes) were higher than the stromal scores (blue boxes), and the cumulative proportion curves, generated using the geom_step() function from the ggplot2 package, show that immune scores (red lines) were consistently located to the right of stromal scores (blue lines) in both ESTIMATE and xCell analyses (The scores for each sample are provided in **Supplementary Tables S3**-**S6**) in the TCGA and Beat AML cohorts. These results illustrated the dominant role of immune infiltration in AML TME. The xCell algorithm was used to estimate the abundance of infiltrating immune cells, and a correlation network was generated (**Supplementary Figure S2I**), presenting the comprehensive associations among different types of immune cells in the TCGA cohort.

**Figure 1:**
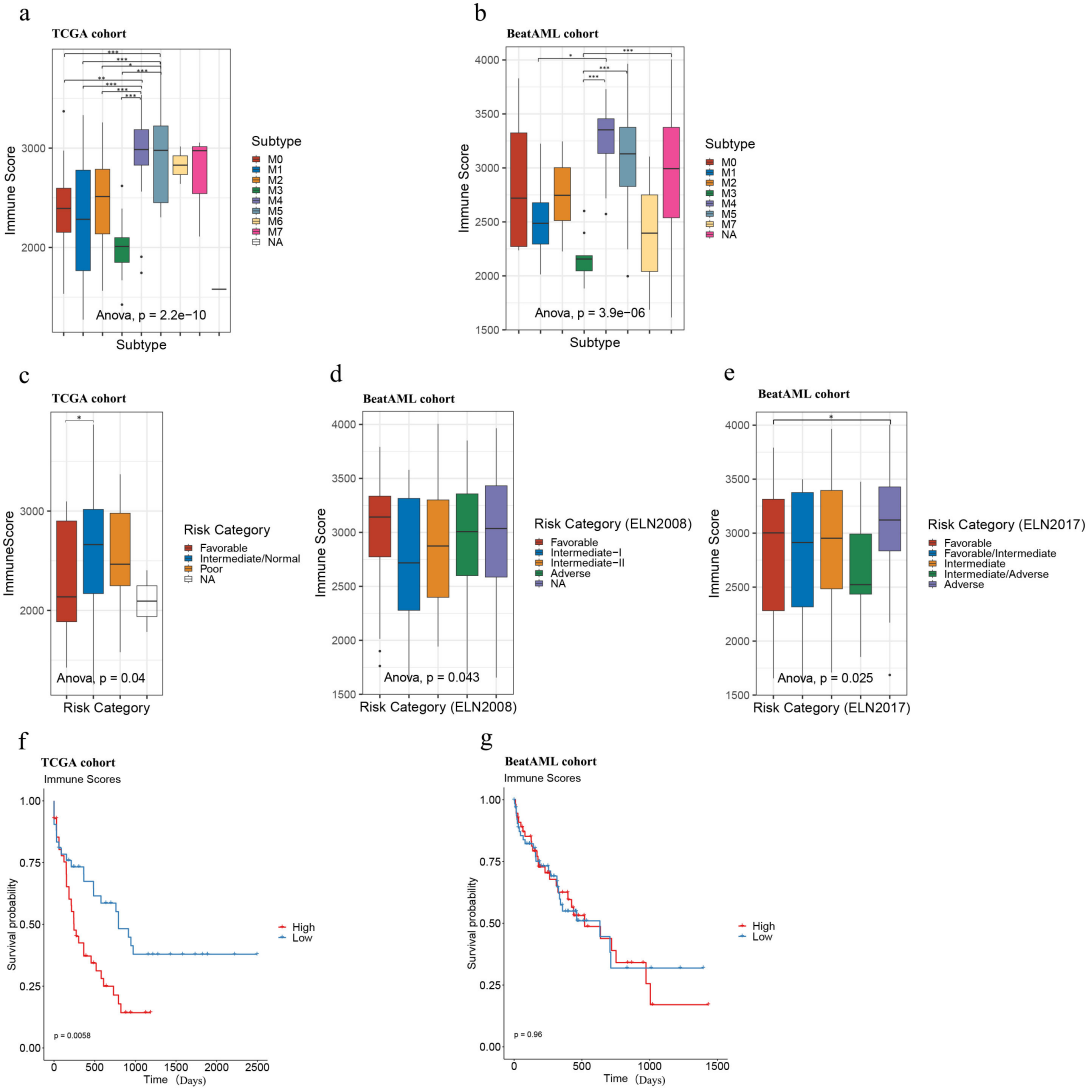
Immune scores of AML samples in the TCGA and Beat AML cohorts. Immune score distributions for AML subtypes **(A, B)**, risk categories **(C–E)**, and survival analysis of high and low immune score samples in TCGA (n = 173; immune scores-high: top 25% samples, n = 43; bottom 25% samples, immune scores-low: n = 43) **(F)** and Beat AML (n = 255; immune scores-high: top 25% samples, n = 64, immune scores-low: bottom 25% samples, n = 64) **(G)** cohorts. Asterisks indicate statistical significance (*p < 0.05; **p < 0.01; ***p < 0.001).

Subsequently, we analyzed the distribution of immune scores among AML subtypes across both cohorts and found a significant association between immune scores and AML subtypes (ANOVA: the TCGA cohort: *p* = 2.2e-10; the Beat AML cohort: *p* = 3.9e-6). The following Tukey’s HSD test further revealed that samples of the M4 and M5 subtypes tended to display exhibited consistently higher immune scores compared to other subtypes, while samples of the M3 subtype showed comparatively lower immune scores in both cohorts (**Figures 1A, B**).

Also, we plotted the distribution of immune scores according to risk categories in the TCGA and Beat AML cohorts. As illustrated in **Figures 1C–E**, although the immune scores were significantly different among different risk categories in both TCGA and Beat AML cohorts according to ANOVA (the TCGA cohort: *p* = 0.04; the Beat AML cohort (ELN2008): *p* = 0.043, Beat AML cohort (ELN2017): *p* = 0.025), the distribution preferences varied. Further analysis with Tukey’s HSD test revealed that, in the TCGA cohort, samples classified as favorable tended to have lower immune scores compared to those classified as intermediate/normal (**Figure 1C**), consistent with Haiment Yang et al.’s research (41). On the other hand, in the Beat AML cohort, Tukey’s HSD test indicated that no significant differences between paired groups based on 2008 European Leukemia Net (ELN) recommendations (**Figure 1D**), while samples classified as the adverse group exhibited relatively higher immune scores compared to the favorable group based on 2017 ELN recommendations (**Figure 1E**). Therefore, we cannot make assumptions about risk levels based solely on immune scores in AML samples.

To explore the potential association of overall survival (OS) with immune scores, we performed Kaplan-Meier survival analysis between immune score-high and low groups in both cohorts. Immune scores were ranked in descending order, with the top 25% classified as ‘immune score-high’ and the bottom 25% as ‘immune score-low.’ In the TCGA cohort, higher immune scores were significantly associated with poorer prognosis (*p* = 0.0058) (**Figure 1F**; **Supplementary Table S7**), which is consistent with the findings of Haiment Yang et al. (41). However, in the Beat AML cohort, no statistically significant differences in overall survival (OS) were observed between the immune score-high and -low groups (**Figure 1G**; **Supplementary Table S8**) (*p* = 0.96). These findings demonstrate the limitations of the prognostic value of immune scores in AML and suggest the need for additional biomarkers to fully predict prognosis in AML.

### Subgroups defined by AML-specific IRGs possess significant prognostic value in AML

We studied differences in immune landscapes between AML samples and normal WB samples using the TCGA-GTEx cohort, which includes 173 AML samples from the TCGA cohort and 337 normal WB samples from the GTEx cohort. After applying cut-off criteria of padj < 0.01 and |log2(Foldchange)| > 1, 911 genes among the 2483 IRGs downloaded from ImmPort (please see the “Materials and methods” section for details) were differentially expressed between AML and normal WB samples in the TCGA-GTEx cohort (**Supplementary Figure S3A**, **Supplementary Table S9**).

**Figure 2:**
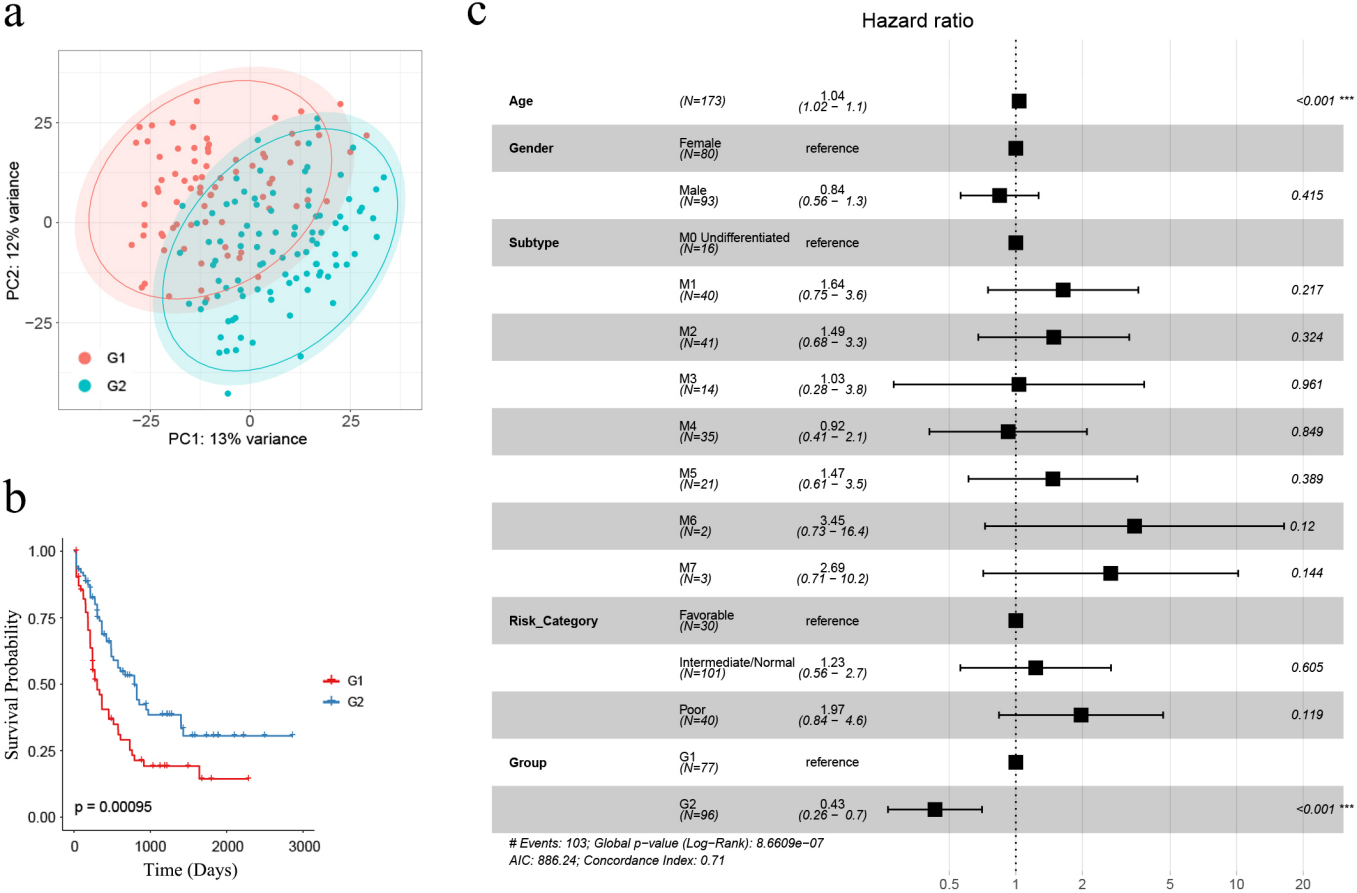
The identification of AML-specific IRGs and corresponding G1, G2 subgroups. **(A)** PCA analysis demonstrated that AML samples classified as G1 and G2 subgroups were roughly separated as two groups by the expression profiles of 850 AML-specific genes; **(B)** Kaplan-Meier survival analysis revealed significantly extended overall survival for G2 subgroup samples (n = 173; G1: n = 77, G2: n = 96) (log-rank test, *p* = 0.00095); **(C)** The multivariate Cox regression showed the subgroups as an independent risk factor, with “G2” associated with a favorable prognosis (*p* < 0.001). Asterisks indicate statistical significance (***p < 0.001).

We aimed to classify AML samples into distinct immune groups based on the 911 AML-specific IRGs. Initially, we filtered the 911 genes by retaining only the 850 genes whose count was greater than 0 in at least 20% (35/173) of the AML samples in the TCGA cohort (n = 173). Subsequently, we performed consensus clustering based on the expression matrix of these 850 AML-specific IRGs. With the range 2 to 10, we selected k=2 for our study based on several observations (42–44). When k=2, we observed that intragroup correlations were high while intergroup correlations were low (**Supplementary Figure S3B**). Additionally, the cluster-consensus scores at each k showed high values for k=2, indicating strong stability of the two clusters (**Supplementary Figure S3C**). These findings suggest that dividing the samples into two clusters based on the 850 AML-specific IRGs is well-supported. Figures illustrating the cluster-consensus scores, cumulative distribution function (CDF), and Delta area plot are provided in the **Supplementary Files** as **Supplementary Figures S3C–E**, which resulted in the separation of the TCGA cohort (n = 173) into two immune subgroups, namely G1 (n = 77) and G2 (n = 96) (**Supplementary Table S10**). According to the results of PCA analysis, classifying AML patients as G1 or G2 by these 850 AML-specific genes could roughly divide the patients into two parts, and this further confirmed two remarkably different subtypes (**Figure 2A**).

Kaplan-Meier survival analysis was performed between G1 and G2, revealing that G1 had a significantly poorer prognosis compared to G2 (n = 173; G1: n = 77, G2: n = 96) (*p* = 0.00095, **Figure 2B**). To evaluate whether G1 and G2 grouping could significantly affect AML prognosis as an independent risk factor, we performed multivariate Cox regression analysis based on clinical characteristics age, gender, subtype, risk category and group. The results revealed that older age was associated with a worse prognosis (HR = 1.04, 95% CI = 1.02 –1.1, *p* < 0.001) while G2-grouping (HR = 0.43, 95% CI = 0.26 – 0.7, *p* < 0.001) was correlated with a more favorable prognosis (**Figure 2C**).

### Establishment and validation of a grouping prediction model

**Figure 3:**
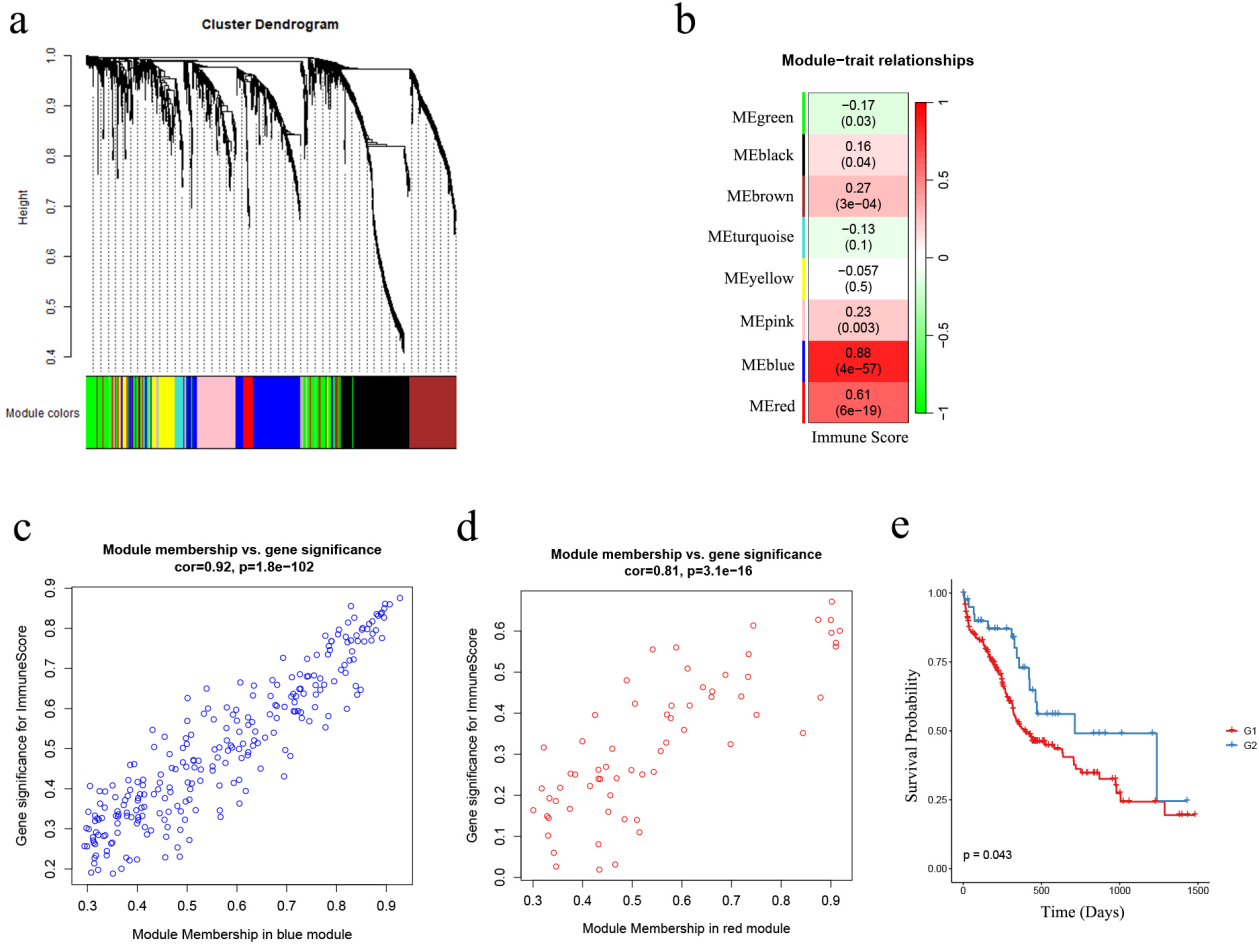
The identification of AML-immune-specific modules and key-AML-IRGs. **(A)** Construction of a scale-free co-expression network using the IRGs expression matrix of AML samples and their immune scores through WGCNA; **(B)** Generation of eight gene modules; designation of blue and red modules as “AML-immune-specific” due to their strong correlation with sample immune scores (|r| = 0.61, p < 0.001; |r| = 0.88, p < 0.001, respectively); **(C, D)** Correlation analysis between gene significance (GS) and module membership (MM) within the blue and red modules; **(E)** Kaplan-Meier analysis demonstrated the sustained favorable prognosis of G2 samples compared to G1 samples in the validation cohort (the Beat AML cohort) after subgroup predicting (n = 255; G1: n = 214, G2: n = 41) (log-rank test, *p* = 0.043). WGCNA, weighted gene co-expression network analysis; IRG, immune-related genes.

To better understand in the total of 2483 IRGs, which ones are associated with immune status in AML patients, we performed WGCNA based on the IRGs expression matrix and immune score levels of 173 AML samples from the TCGA cohort. We constructed a scale-free co-expression network (**Figure 3A**) after excluding two outlier samples (**Supplementary Figure S4A**). Eight gene modules were generated with a power of 4 (**Supplementary Figure S4B**; **Figure 3B**). Among these modules, the blue and red modules showed the strongest correlation with sample immune scores (|r| = 0.61, *p* < 0.001; |r| = 0.88, *p* < 0.001, respectively) and were considered as “AML-immune-specific” modules (**Figure 3B**; **Supplementary Tables S11**, **S12**).

Using GS and MM measures, we identified “key-AML-IRG” by selecting IRGs in the two “AML-immune-specific” modules. As shown in **Figures 3C, D**, IRGs in both modules exhibited strong correlations between GS and MM (blue module IRGs: cor = 0.92, *p* < 0.001; red module IRGs: cor = 0.81, *p* < 0.001), denoting that these 314 IRGs, which were significantly associated with immune score, were also crucial elements of immune-score associated modules. Therefore, we considered these 314 IRGs as “key-AML-IRGs” in AML patients.

Utilizing the AutoGluon algorithm, we constructed an AML subgroup predicting model based on the expression matrix of the 314 key-AML-IRGs (Please refer to **Supplementary Table S13** to see the model details). AML samples from Beat AML samples were classified as G1 or G2 subgroups using this model. Survival analysis revealed that in the Beat AML cohort, samples classified as G2 group had a more favorable prognosis compared to G1 (*p* = 0.043, **Figure 3E**), demonstrating the prognostic value of the AML subgroup predicting model.

### Overall comparison between G1 and G2

We compared the G1 and G2 subgroups in the TCGA and Beat AML cohorts to enhance our understanding of the two subgroups of their biological and pathological characteristics.

**Figure 4:**
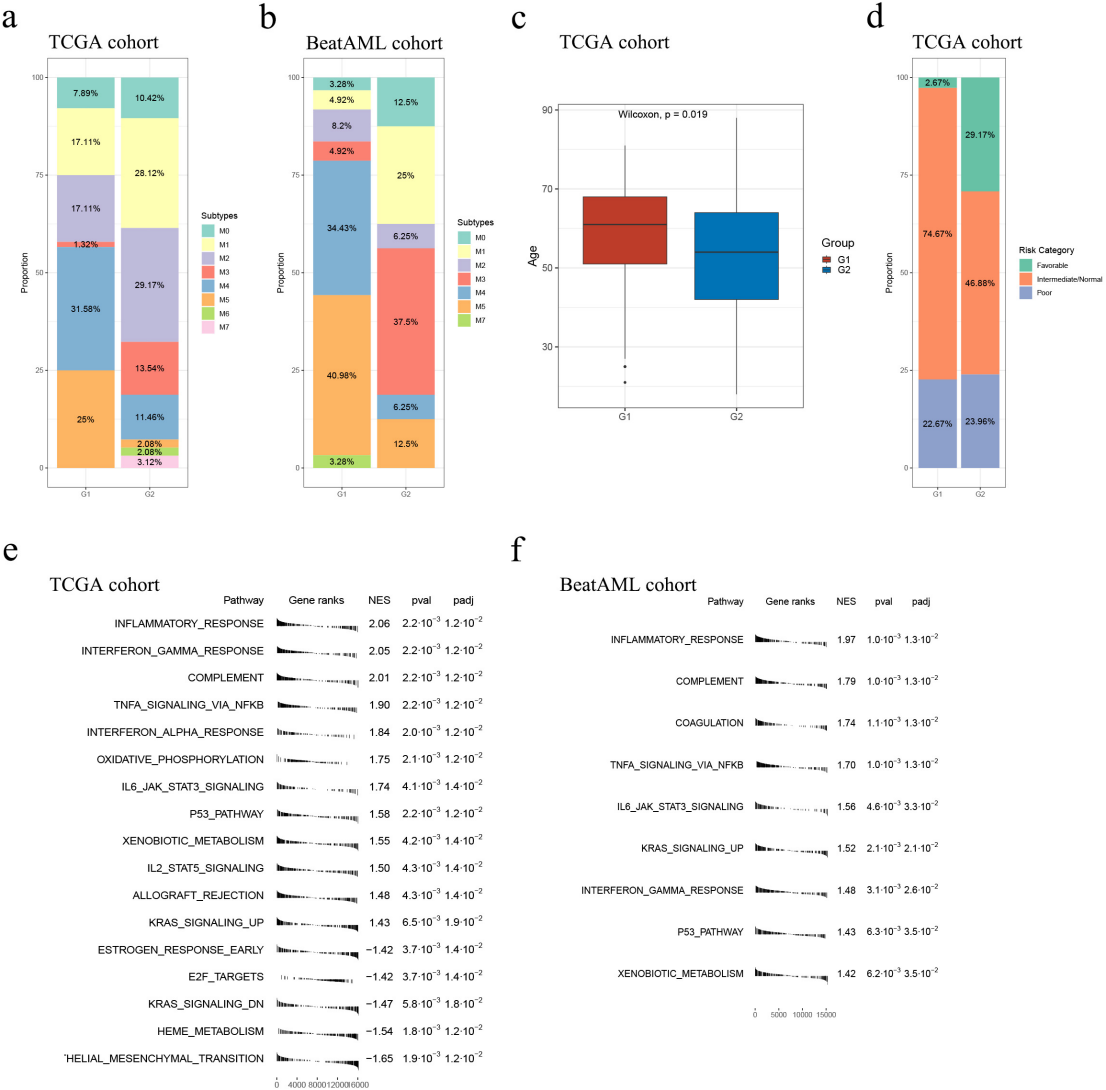
Overall comparison between G1 and G2. Subtype distribution in the TCGA **(A)** and Beat AML **(B)** cohorts across G1 and G2 subgroups; **(C)** In the TCGA cohort, the G1 subgroup consisted of relatively older individuals compared to the G2 subgroup; **(D)** Comparison of risk category distribution between the G1 and G2 subgroups in the TCGA cohort; GSEA analyses conducted between the G1 and G2 subgroups in the TCGA **(E)** and Beat **(F)** AML cohort.

In both cohorts, G1 had a higher proportion of samples belonging to the M4 or M5 subtypes, whereas G2 had a higher proportion of samples belonging to the M1 or M3 subtypes (the TCGA cohort: *p* = 2.64e-7, the Beat AML cohort: *p* = 0.0002; **Supplementary Tables S14**, **S15**; **Figures 4A, B**). Moreover, within the TCGA cohort, G2 exhibited younger ages and a greater proportion of samples classified as “Favorable”, consistently indicating its correlation with a more favorable prognosis compared to G1 (*p* = 0.019, *p* = 0.005, respectively; **Table 2**, **Figures 4C, D**). However, no specific distribution preferences for “age” or “risk category” were observed between G1 and G2 in the Beat AML cohort (**Table 3**). Additionally, G2 showed a higher percentage of BM blast counts compared to G1 (*p* < 0.001) in the Beat AML cohort, while no significant difference in the BM blast counts between the two subgroups was observed in the TCGA cohort (**Tables 2**, **3**).

**Table 2:** The baseline characteristics of G1 and G2 subgroups in the TCGA cohort.

Variables		G1 (n = 77)	G2 (n = 96)	P value
**Gender**	Male	41	44	4.14E-01
	Female	36	52	
**Median age (range), y**		61(21-81)	54(18-88)	**1.86E-02**
**Median bone marrow blast (rage), %**		37(0-98)	34.5(0-97)	7.48E-01
**FAB Subtypes**	M0	6	10	**2.64E-07**
	M1	13	27	
	M2	13	28	
	M3	1	13	
	M4	24	11	
	M5	19	2	
	M6	0	2	
	M7	0	3	
	NA	1	0	
**Risk Category**	Favorable	2	28	**5.12E-03**
	Intermediate/Normal	56	45	
	Poor	17	23	
	NA	2	0	

The bolded p-values in the table indicate statistically significant differences in the baseline characteristics between the G1 and G2 subgroups (p < 0.05). NA, Not Available.

**Table 3:** The baseline characteristics of G1 and G2 subgroups in the Beat AML cohort.

Variables		G1 (n = 214)	G2 (n = 41)	P value
**Gender**	Male	105	14	1.13E-01
	Female	109	27	
**Median age (range), y**		61(2-87)	56(7-85)	5.17E-01
**Median bone marrow blast (rage), %**		69(1-98)	85.55(21-97)	**3.47E-04**
**FAB Subtypes**	M0	2	2	**2.00E-04**
	M1	3	4	
	M2	5	1	
	M3	3	6	
	M4	21	1	
	M5	25	2	
	M6	0	0	
	M7	2	0	
	NA	153	25	
**Risk Category (ELN2008)**	Favorable	48	11	4.78E-01
	Intermediate-I	34	6	
	Intermediate-II	39	6	
	Adverse	45	11	
	NA	48	7	
**Risk Category (ELN2017)**	Favorable	70	16	2.85E-01
	Favorable/Intermediate	9	1	
	Intermediate	63	10	
	Intermediate/Adverse	3	3	
	Adverse	69	11	

The bolded p-values in the table indicate statistically significant differences in the baseline characteristics between the G1 and G2 subgroups (p < 0.05). NA, Not Available.

A total of 1605 and 1715 DEGs were identified between the G1 and G2 subgroups in the TCGA and Beat AML cohorts, respectively (**Supplementary Tables S14**, **S15**). Instead of simply intersecting the DEGs from both cohorts, we implemented the RRA algorithm to address substantial heterogeneity and potential errors arising from differences in technological platforms and statistical methods. This approach allowed us to select 154 robust DEGs with an RRA score < 0.05, as these genes were more likely to be true DEGs based on their |log2(FoldChange)| values (**Supplementary Table S16**). **Supplementary Figures S5**, **S6** display the 154 robust DEGs between the two groups in the TCGA and Beat AML cohorts. Please note that the number of genes shown in each heatmap is slightly less than 154, with 139 and 131 genes displayed, respectively. Subsequently, we performed enrichment analysis of drugs/compounds and identified 317 drugs/compounds that may exhibit differential effectiveness in G1 and G2, and the top 20 drugs/compounds were 9cRA, arsenite, vinylidene chloride, magnesium, Betamethasone-d5, panobinostat, phorbol acetate myristate, Diethylhexyl Phthalate, sodium bichromate and ethylnylestradiol (**Table 4**; **Supplementary Table S17**).

**Table 4:** TOP20 drugs/compounds exhibiting differential effectiveness in G1 and G2 subgroup (based on the expression data of 154 robust DEGs).

Category	ID	Name	Source	p-value	q-value FDR B&H	Hit Count in Query List	Hit Count in Genome	Hit in Query List
Drug	CID000005538	9cRA	Stitch	3.97E-08	**2.59E-04**	33	1735	COL2A1,CDKN2B,ONECUT2,CNTN4,LAMB4,CES1,PDK4,CYP26A1,CLEC7A,POU1F1,POU3F3,LAMC3,ADAMTS5,RUNX1T1,SIX3,EDN3,KRT23,PPARGC1A,HOXB6,THBS1,HOXB8,PI3,CADM1,IGF2,SLC10A2,CD14,BRINP2,CCR1,FGF10,PAX2,NCR2,HBE1,CX3CR1
Drug	ctd:C015001	arsenite	CTD	1.15E-07	**4.60E-04**	32	1723	COL2A1,NDNF,NMNAT2,FAM163A,PDK4,PENK,POU3F3,GRIK3,GPC6,HNMT,AQP9,LEP,CD163,TMTC1,HOXB6,THBS1,HOXB8,HOXC10,CD14,IL1R2,VCAN,PITX1,NR0B1,MKX,KCNN2,DGKI,CCR1,MAFB,SNAP25,MLPH,FOXF2,FOXC2
Drug	ctd:C029297	vinylidene chloride	CTD	1.58E-07	**5.24E-04**	29	1476	NDNF,MYO7A,NCF1,CLEC7A,MECOM,B3GALT5,POU3F3,CAMK1,EPB41L3,SLC7A7,PTGFR,POSTN,DEFB1,PPARGC1A,GDA,TMTC1,SERPINA1,CADM1,SLC10A2,FBP1,SLC11A1,CD14,KCNE1,ATRNL1,IFI30,C2,CCR1,IRX2,MLPH
Drug	CID000000888	magnesium	Stitch	2.21E-07	**5.94E-04**	27	1325	COL2A1,PRSS2,LAMB4,NMNAT2,HK3,MYO7A,PDK4,LAMC3,GRIK3,REN,RASL12,LGSN,AQP9,IGF2,FBP1,SLC11A1,KCNE1,SMPDL3A,VCAN,KCNN2,DGKI,C2,SNAP25,MLPH,MARCO,PRL,EPHA3
Drug	CID000003003	Betamethasone-d5	Stitch	2.76E-07	**6.12E-04**	27	1340	COL2A1,PRSS2,LAMB4,SPOCK1,PDK4,CYP26A1,PENK,HPSE2,LAMC3,REN,LGSN,LEP,CD163,EDN3,CD300C,POSTN,DEFB1,PPARGC1A,SERPINA1,IGF2,SLC10A2,CD14,SCGB3A2,IL1R2,VCAN,HBE1,PRL
Drug	ctd:C496932	panobinostat	CTD	8.78E-07	**1.20E-03**	26	1333	GABRB2,ONECUT2,NMNAT2,FAM163A,MECOM,POU3F3,GRIK3,RUNX1T1,EPB41L3,SIX3,GPC6,HNMT,IGF2,OLFM3,SCGB3A2,SMPDL3A,UNC5C,IFI30,MAFB,PAX2,SNAP25,IRX2,PID1,EPHA3,WDR72,FOXC2
Drug	CID000004792	phorbol acetate myristate	Stitch	6.89E-06	**4.01E-03**	25	1399	COL2A1,PRSS2,LILRB3,CES1,PENK,LILRA6,POU1F1,HPSE2,CAMK1,PTAFR,PTGFR,CD163,EDN3,KRT23,PPARGC1A,PI3,CD14,IL1R2,SMPDL3A,VCAN,DGKI,TRPV4,CCR1,SNAP25,PRL
Drug	ctd:D004051	Diethylhexyl Phthalate	CTD	1.10E-04	**2.19E-02**	25	1654	NMNAT2,HK3,PDK4,CYP26A1,CLEC7A,REN,LEP,LGALS2,KRT23,PPARGC1A,GDA,HOXB6,THBS1,IGF2BP1,CADM1,IGF2,SLC10A2,KCNE1,NR0B1,KCNN2,PAX2,PRL,CX3CR1,EPHA3,WDR72
Drug	ctd:C016104	sodium bichromate	CTD	1.98E-04	**3.02E-02**	24	1615	CDKN2B,PRSS2,HK3,SIGLEC9,CYP26A1,CLEC7A,B3GALT5,CAMK1,SLC7A7,CD163,LGALS2,TCF23,GDA,SERPINA1,SLC10A2,FBP1,CD14,SMPDL3A,NR0B1,KCNN2,IFI30,CCR1,RAB39A,MAFB
Drug	CID000003285	ethylnylestradiol	Stitch	1.07E-05	**4.85E-03**	23	1251	COL2A1,CYP26A1,PENK,POU1F1,REN,TYMP,LGSN,AQP9,LEP,EDN3,KRT23,PPARGC1A,SERPINA1,PI3,IGF2,SCGB3A2,IFI30,CCR1,FGF10,SAGE1,HBE1,PRL,CX3CR1
Drug	CID000145068	nitric oxide	Stitch	3.15E-06	**2.73E-03**	22	1075	CASP5,REN,PPP1R17,PTAFR,SLC7A7,GPC6,LGSN,TLR8,LEP,EDN3,PPARGC1A,THBS1,PI3,SLC7A3,SLC11A1,CD14,IL1R2,VCAN,TLR5,KCNN2,HBE1,PRL
Drug	CID000000813	potassium	Stitch	4.10E-06	**3.22E-03**	21	1008	GABRB2,SPOCK1,PENK,REN,CAMK1,SLC7A7,LEP,EDN3,KCNH6,TCF23,SLC7A3,FBP1,SLC11A1,KCNE1,KCNN2,TRPV4,C2,SNAP25,HBE1,PRL,CX3CR1
Drug	CID000004920	17-isoprogesterone	Stitch	3.83E-05	**1.29E-02**	21	1170	COL2A1,GABRB2,GABRE,CYP26A1,PENK,HPSE2,GRIK3,REN,TYMP,PTGFR,LEP,KRT23,PPARGC1A,THBS1,IGF2,SCGB3A2,IFI30,C2,HBE1,PID1,PRL
Drug	CID000000450	17alpha-estradiol	Stitch	1.75E-04	**2.76E-02**	21	1303	GABRB2,GABRE,PENK,HPSE2,TYMP,LGSN,PTGFR,AQP9,LEP,EDN3,THBS1,SERPINA1,PI3,IGF2,SCGB3A2,IFI30,CCR1,FGF10,SAGE1,PRL,CX3CR1
Drug	ctd:D007545	Isoproterenol	CTD	1.60E-05	**6.64E-03**	20	1015	COL2A1,LILRB3,NCF1,PENK,REN,PTGFR,LEP,CD163,POSTN,KCNH6,DEFB1,TCF23,PPARGC1A,GDA,TMTC1,CD14,KCNE1,VCAN,CCR1,PRL
Drug	CID000001117	sulfate	Stitch	4.24E-04	**3.25E-02**	20	1292	COL2A1,PRSS2,LAMB4,SPOCK1,CES1,HPSE2,LAMC3,GPC6,POSTN,DEFB1,THBS1,CADM1,IGF2,EFNB3,VCAN,HRC,C2,FGF10,MLPH,MARCO
Drug	CID000002900	isocycloheximide	Stitch	1.10E-05	**4.88E-03**	19	905	COL2A1,PRSS2,LAMB4,PENK,CASP5,LAMC3,PTAFR,PI15,LEP,KRT23,THBS1,IGF2,CD14,IL1R2,VCAN,C2,SNAP25,HBE1,PRL
Drug	ctd:D012906	Smoke	CTD	6.08E-05	**1.33E-02**	18	937	OLAH,CDKN2B,IL31RA,SPOCK1,PDK4,SLC7A7,LEP,PPARGC1A,GDA,THBS1,SERPINA1,CD14,IL1R2,EVA1A,VCAN,MARCO,CX3CR1,FOXC2
Drug	ctd:C042720	mercuric bromide	CTD	3.37E-04	**3.20E-02**	18	1076	GABRB2,ONECUT2,PRSS2,PPP1R17,RUNX1T1,EPB41L3,SLC7A7,SIX3,HNMT,PI15,POSTN,THBS1,OLFM3,SMPDL3A,DGKI,MAFB,CDH6,FOXC2
Drug	CID000003715	indomethacin	Stitch	4.20E-06	**3.22E-03**	17	690	PRSS2,LAMB4,HPSE2,LAMC3,REN,PTAFR,PTGFR,EDN3,TMTC1,THBS1,IL1R2,VCAN,TRPV4,C2,IRX2,HBE1,PRL

The bolded q-values (FDR B&H) in the table indicate a statistically significant difference in the effectiveness of the drugs/compounds between the G1 and G2 subgroups (q < 0.05).

We conducted GSEA to compare pathways between the G1 and G2 subgroups using 50 hallmark gene sets from the MSigDB database. The results, depicted in **Figures 4E, F** (please see detailed information in **Supplementary Tables S18**, **S19**), demonstrated that the G2 subgroup exhibited significantly higher activity in various immune processes compared to G1. Specifically, the “inflammatory response” pathway was consistently identified as the most significantly altered pathway in both cohorts. Additionally, several classical tumor-related pathways, such as “TNFα signaling via NFκB”, “oxidative phosphorylation”, “IL6 JAK STAT3 signaling”, “P53 pathway” and “KRAS signaling up”, showed higher activity in G2 in both cohorts (**Figures 4E, F**; **Supplementary Tables S18**, **S19**). Overall, the GSEA results unveiled a less malignant level of samples classified as G2.

### Immune characterization of G1 and G2

Subsequently, we compared the immune characterization of G1 and G2.

**Figure 5:**
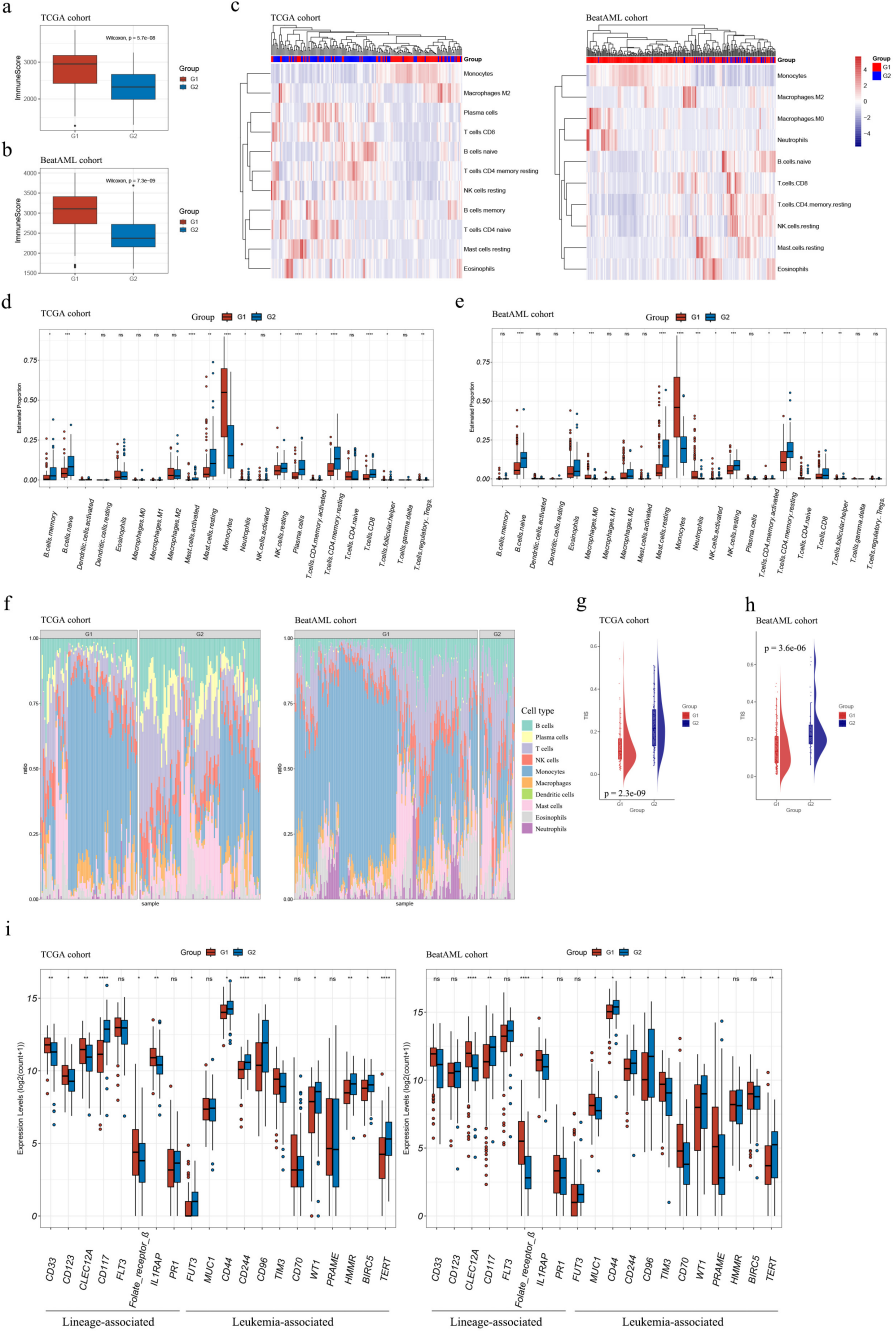
Immune characterization of the G1 and G2 subgroups. **(A, B)** Boxplots demonstrated higher immune scores for G1 subgroup samples compared to G2 subgroup samples in both TCGA **(A)** and Beat AML **(B)** cohorts; **(C)** Estimation of infiltrating immune cell abundances in AML samples from TCGA and Beat AML cohorts; **(D, E)** Abundance profiles of each tumor microenvironment (TME) infiltrating cell within G1 and G2 subgroups in TCGA **(D)** and Beat AML **(E)** cohorts; **(F)** Stacked barplots depicted distinct relative proportions of 22 immune cell types between G1 and G2 subgroups in TCGA and Beat AML cohorts; **(G, H)** TIS significantly increased in G2 subgroups compared to G1 (p < 0.001) in both cohorts; **(I)** The expression levels of AML T-cell-based immunotherapy target antigens in the G1 and G2 subgroups in the TCGA and Beat AML cohorts. Asterisks indicated statistical significance (*p < 0.05; **p < 0.01; ***p < 0.001; ****p < 0.0001); “ns” denoted no significance. TIS, T cell infiltration score.

In both cohorts, G1 exhibited consistently elevated immune infiltration (estimated by immune score) compared to G2 (both p < 0.001; see **Figures 5A, B**).

Our findings about the abundances of infiltrating immune cells using xCell suggested that higher levels of monocytes and macrophages were associated with G1, whereas higher levels of T cells, NK cells, B cells, and mast cells were associated with G2 (**Figure 5C**).

The assessment of abundances of immune cells between the two subgroups using CIBERSORT was consistent with results mentioned above. G1 demonstrated relatively higher cell proportions of monocytes, and G2 showed higher cell proportions of B memory cells, naïve B cells, activated mast cells, resting NK cells, activated and resting CD4+ T memory cells, CD8+ T cells, and T follicular helper cells (**Figures 5D–F** and **Supplementary Tables S20**, **S21**). Moreover, TIS were substantially elevated in G2 compared with G1 in both datasets (*p* < 0.001, **Figures 5G, H**), primarily due to an increase of activated CD4+ memory T cells and CD8+ T cells in G2 (**Figures 5D, E**).

The expression levels of target antigens in AML T-cell-based immunotherapy, as summarized by Naval Daver et al. (13), were compared between the G1 and G2 subgroups. **Figure 5I** illustrates that the trends of antigen expression levels were consistent in the TCGA and Beat AML cohorts. Notably, among the antigens with statistically expression differences in both cohorts, CD117, CD44, CD244, CD96, WT, and TERT exhibited higher expression levels in G2 compared to G1; while CLEC12A, Folate receptor β, IL1RAP, and TIM3 showed higher expression levels in G1 compared to G2. It should be mentioned that in **Figure 5I**, the expression levels of CD44 were presented instead of CD44v6. These suggested that different antibody choices may be warranted in immunotherapy for samples classified into the G1 or G2 subgroups.

### Mutational landscapes between the subgroups in the TCGA and Beat AML cohorts

Given the strong association between immune infiltration and mutations in tumors, additionally, the high frequencies of mutations in multiple genes in AML patients and their relationship with AML prognosis, we conducted an investigation into the mutational landscape of G1 and G2 in both cohorts.

**Figure 6:**
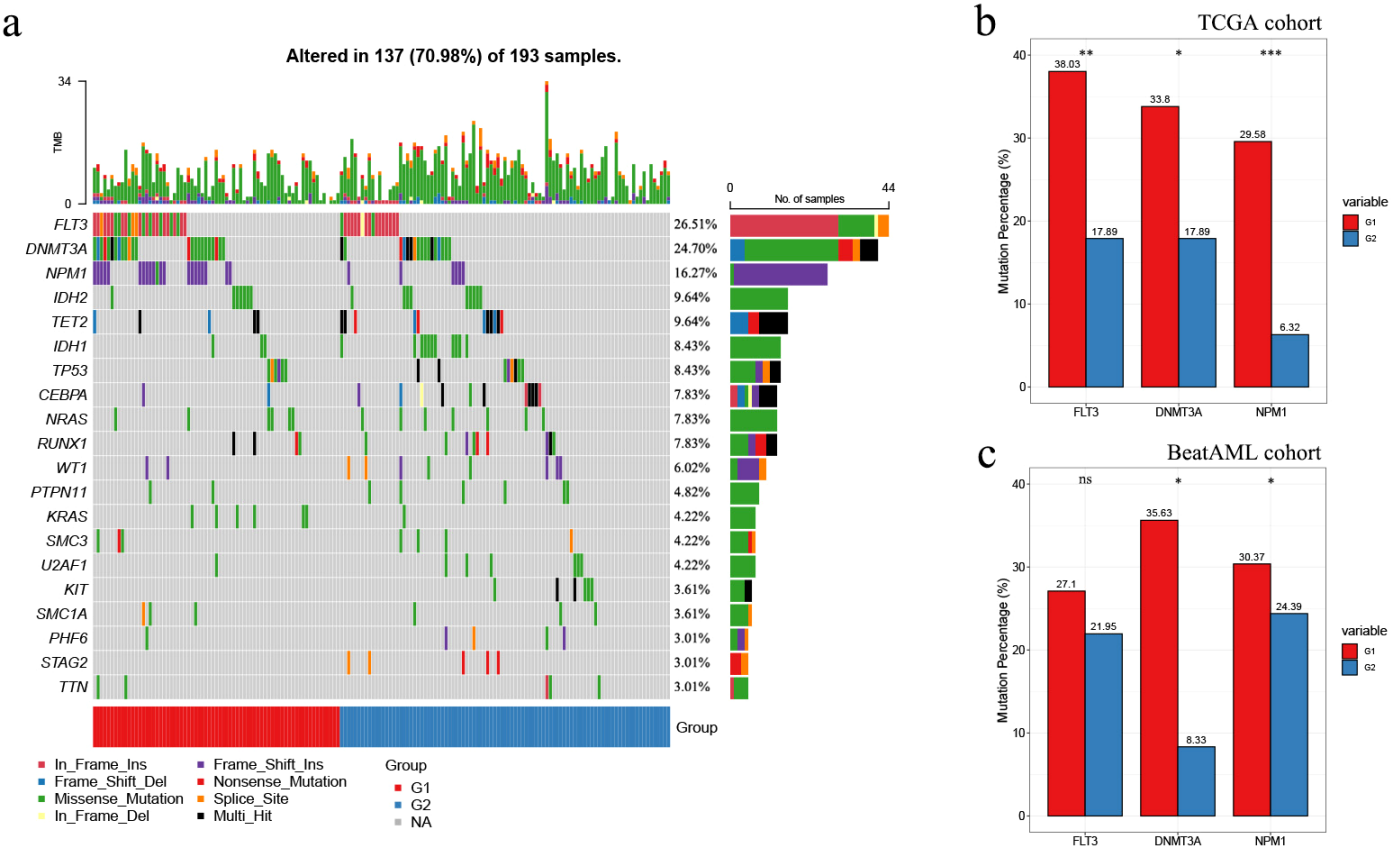
Comparison of mutational landscapes between the subgroups in the TCGA and Beat AML cohorts. **(A)** Oncoplot displaying the top 20 frequently mutated genes within G1 and G2 subgroups in the TCGA cohort; **(B, C)** Proportions of FLT3, DNMT3A, or NPM1 mutated samples in TCGA **(B)** and Beat AML **(C)** cohorts. Asterisks indicated statistical significance (*p < 0.05; **p < 0.01; ***p < 0.001; “ns” denoted no significance.

Oncoplot displaying the ranking of the top 20 frequently mutated genes in G1 and G2 was generated for G1 and G2 in the TCGA cohort. FLT3, DNMT3A, and NPM1 emerged as the top 3 genes with the highest mutation frequencies across all AML samples. These genes exhibited a mutation preference in G1 compared to G2 (p-values of 0.006, 0.030, and < 0.001, respectively; **Table 5**; **Figures 6A, B**). In addition, it is worth mentioning that mutated FLT3 (ITD) and mutated NPM1 are also leukemia-specific target antigens in AML, which are associated with CD8+ T-cell responses (13).

**Table 5:** Mutation frequencies of FLT3, DNMT3A and NPM1 in the G1, G2 subgroups in the TCGA and Beat AML cohorts.

Cohorts		FLT3	DNMT3A	NPM1
**TCGA**	**G1**	27/71 (38.03%)	24/71 (33.80)	21/71 (29.58)
	**G2**	17/95 (17.89%)	17/95 (17.89%)	6/95 (6.32%)
	**Overall**	44/166(26.51%)	41/166 (24.70%)	27/166 (16.27%)
	**P value (G1 vs. G2)**	**0.006**	**0.030**	**<0.001**
**Beat AML**	**G1**	58/214 (27.10%)	31/87 (35.63%)	65/214 (30.37%)
	**G2**	9/41 (21.95%)	2/24 (8.33%)	10/41 (24.39%)
	**Overall**	67/255 (26.27%)	33/111 (29.73%)	75/255 (29.41%)
	**P value (G1 vs. G2)**	0.622	**0.019**	**0.035**

The bolded p-values in the table indicate statistically significant differences in mutation frequencies between the G1 and G2 subgroups (p < 0.05).

As for the Beat AML cohort, according to the gene mutation data from the clinical information (20, 21), FLT3, DNMT3A, and NPM1 also showed high mutation frequencies. Similarly, these genes exhibited higher mutation preferences in G1. However, the statistical difference was only observed for DNMT3A and NPM1 in the Beat AML cohort (p values of 0.019 and 0.035, respectively; **Table 5**; **Figure 6C**).

### Drug sensitivity analyses in the Beat AML cohort

**Figure 7:**
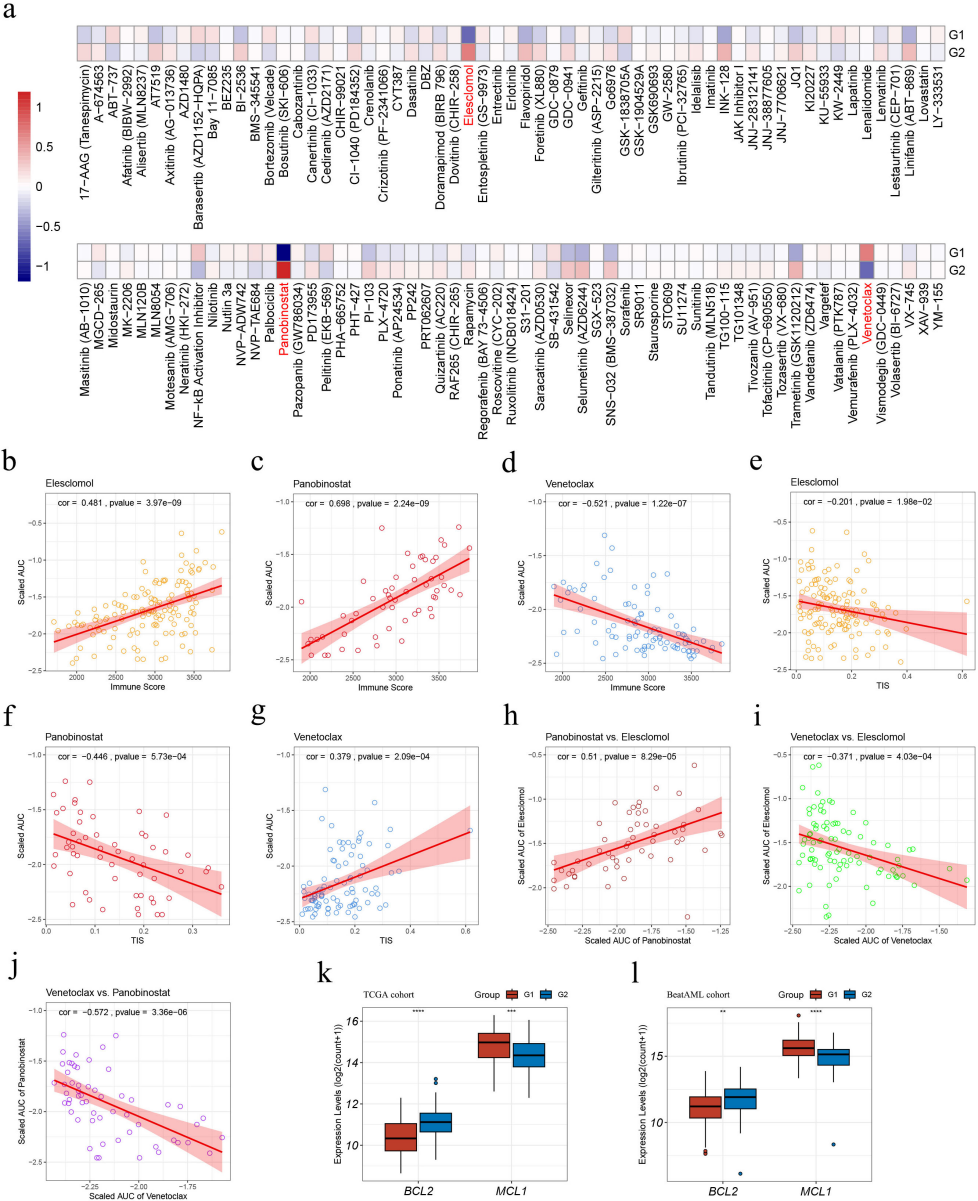
Drug sensitivity analyses within the G1 and G2 subgroups in the Beat AML cohort. **(A)** Heatmap illustrating treatment responses of the subgroups to various small-molecule inhibitors based on Beat AML ex vivo drug screen data. Drug responses were quantified by scaled AUC values, where blue signified sensitivity and red denoted resistance; **(B–D)** Pearson correlation analyses assessing associations between sample immune scores and scaled AUC values of elesclomol **(B)**, panobinostat **(C)**, and venetoclax **(D)**; **(E–G)** Pearson correlation analyses evaluating correlations between sample TIS and scaled AUC values of elesclomol **(E)**, panobinostat **(F)**, and venetoclax **(G)**; **(H–J)** Pearson correlation analyses on pairs of scaled AUC values: **(H)** elesclomol and panobinostat, **(I)** elesclomol and venetoclax; **(J)** panobinostat and venetoclax; **(K–L)** Expression levels of BCL2 and MCL1 in the TCGA **(K)** and Beat AML **(L)** cohorts. AUC, Area under the dose-response curve; TIS, T cell infiltration score. Asterisks indicate statistical significance (**p < 0.01; ***p < 0.001, ****p < 0.0001).

We predicted the responses of G1 and G2 AML samples to 122 small-molecule inhibitors according to the ex vivo drug sensitivity data from the Beat AML cohort (20, 21) (**Figure 7A**; **Supplementary Table S22**). G1 exhibited a higher sensitivity to elesclomol and panobinostat and a less sensitivity to venetoclax (|FoldChange| > 1.5, p < 0.05, **Figure 7A**).

The application of Pearson correlation analysis to the drug scaled AUC values and sample immune scores revealed that, the scaled AUC values of elesclomol and panobinostat displayed a positive correlation with sample immune scores (cor = 0.481, *p* = 3.97e-9; cor = 0.698, *p* = 2.24e-9, respectively) (**Figures 7B, C**), whereas the scaled AUC values of venetoclax and sample immune scores were negatively correlated (cor = -0.521, *p* = 1.22e-7) (**Figure 7D**). These suggest that higher sample immune infiltration levels are associated with resistance to elesclomol and panobinostat, while sensitivity to venetoclax.

Moreover, apart from a weak negative correlation between the scaled AUC values of elesclomol and sample TIS (cor = -0.201, *p* = 1.98e-2) (**Figure 7E**), the scaled AUC values of panobinostat were negatively correlated with sample TIS (cor = -0.446, *p* = 5.73e-4) (**Figure 7F**), while those of venetoclax displayed a positive correlation with sample TIS (cor = -0.379, *p* = 2.09e-4) (**Figure 7G**). These findings associated higher TIS values with sensitivity to panobinostat and resistance to venetoclax.

Pearson correlation analysis on pairs of scaled AUC values among the three mentioned drugs revealed positive correlations between the sensitivity of AML samples to elesclomol and panobinostat (cor = -0.51, *p* = 8.29e-5) (**Figure 7H**), and negative correlations between the sensitivity to elesclomol and venetoclax (cor = -0.371, *p* = 4.03e-4) (**Figure 7I**), as well as to panobinostat and venetoclax (cor = -0.572, *p* = 3.36e-6) (**Figure 7J**). These findings suggest that elesclomol or panobinostat, particularly panobinostat due to its stronger correlation with sensitivity to venetoclax, may serve as potential alternative treatment options for AML patients who are resistant to venetoclax.

Additionally, in both cohorts, G1 showed a significantly lower BCL2 expression and higher MCL1 expression, as depicted in **Figures 7K, L**.

### AML subgroups correlate with cellular hierarchies

**Figure 8:**
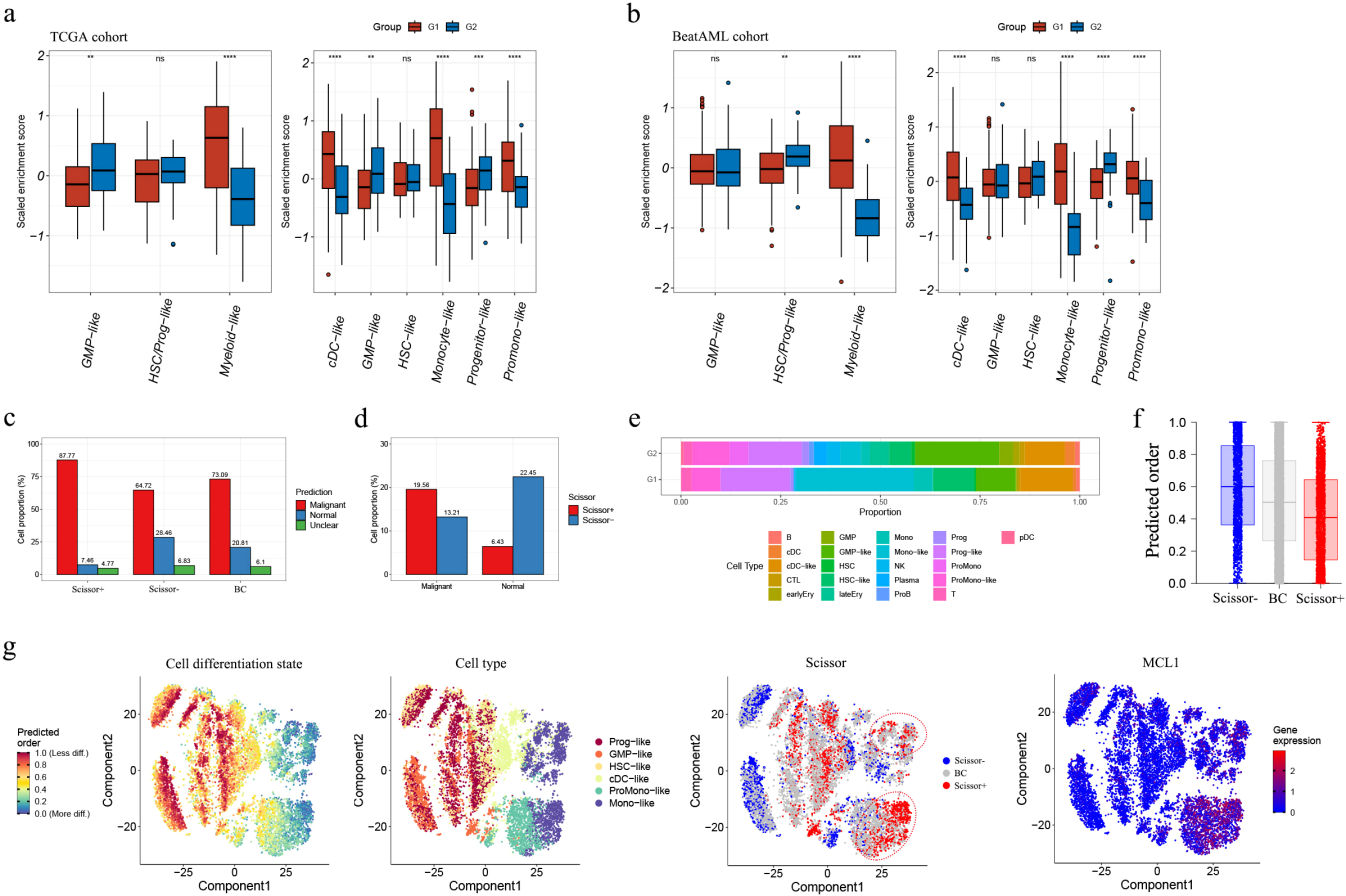
Integrated analyses with scRNA-Seq dataset GSE116256. **(A, B)** Estimation of enrichment of malignant cell types within G1 and G2 subgroups in the TCGA **(A)** and Beat AML **(B)** cohorts; **(C)** Barplots illustrating proportions of malignant/normal cells in Scissor+ and Scissor- cells; **(D)** Barplots showing proportions of Scissor+/Scissor- cells in malignant and normal cell populations; **(E)** Stackplot depicting the proportions of malignant and normal cell types in G1-featured (Scissor+) and G2-featured (Scissor-) cells; **(F)** Differentiation state comparison of malignant Scissor+ and Scissor- cells (higher predicted order implied less differentiation); **(G)** t-SNE plots displaying the differentiation state, the distribution of cell type, Scissor label and MCL1 expression. t-SNE, t-distributed stochastic neighbor embedding. Asterisks indicate statistical significance (**p < 0.01; ***p < 0.001; ****p < 0.0001).

To explore the relationship of AML subgroups with cellular hierarchies, we referred to single-cell RNA-Seq (scRNA-Seq) data reported by Galen et al. (23) to pinpoint gene signatures of diverse differentiation states. Scaled enrichment scores for each cell type were calculated among samples of the TCGA and Beat AML cohorts. As shown in **Figures 8A, B**; **Supplementary Tables S23**, **S24**, G1 and G2 enriched distinct cell-type signatures. In both cohorts, G1 showed significantly higher cell-type enrichment scores for cDC-like, monocyte-like and promonocyte-like cells and lower cell-type enrichment scores for progenitor-like cells, suggesting a more monocytic phenotype for the G1 samples.

Next, we used Scissor to label G1- and G2- featured cells as Scissor+ and Scissor- cells, which included 2,560 and 2,344 cells, respectively. According on the refined prediction results for each cell in the original study (22), Scissor+ cells exhibited a relatively higher proportion of malignant cells and a lower proportion of normal cells compared to Scissor- cells (**Figure 8C**); within the malignant cell population, the Scissor+ cells were more abundant than the Scissor- cells, whereas the opposite was observed in the normal cell population (**Figure 8D**) (**Supplementary Table S25**). Also, we analyzed the distribution of different cell types in both groups based on the cell type annotation provided in the original study (23). The proportions of each cell type in the two groups were calculated (**Figure 8E**). Notably, Scissor+ cells exhibited higher proportions of cDC-like, HSC-like, monocyte-like, and monocyte cells compared to Scissor- cells. Scissor- cells had higher proportions of GMP and GMP-like cells, as well as various normal cell types including cDC, cytotoxic T-lymphocyte (CTL), early erythroid, HSC, late erythroid, NK, plasma, proB, progenitor, promonocyte, and pDC cells. Interestingly, among the normal cells, all the Scissor labeling plasma and late erythroid cells were categorized as Scissor-, and no Scissor+ cells were detected.

These findings indicate high malignancy and a more monocytic phenotype in Scissor+ cells, whereas less malignancy and a more immune-activated phenotype in Scissor- cells. These findings were approximately consistent with our cell type analyses in the bulk cohorts (namely TCGA and Beat AML), as mentioned above.

Subsequently, the differentiation states of both Scissor+ and Scissor- malignant cells were analyzed. As shown in **Figure 8F**, the total differentiation state of the malignant Scissor+ cells was found to be higher compared to the malignant Scissor- cells. Moreover, the more differentiated malignant Scissor+ cells exhibited predominant proportions of monocyte-like cells, characterized by a higher expression level of MCL1 (**Figure 8G**).

Next, we categorized the samples as G1-featured or G2-featured based on their Scissor+ or Scissor- cell proportions, utilizing two criteria: 1) The combined number of Scissor+ and Scissor- cells should constitute more than 20% of the total detected cells in each sample, and 2) The FoldChange of Scissor+/Scissor- (or Scissor-/Scissor+) cell proportions in the sample must exceed 1.5 (|log2(FoldChange)| > 0.59). Consequently, we identified five G1-featured samples (AML870, AML419A, AML556, AML328, and AML921A) and four G2-featured samples (AML916, AML707B, AML1012, and AML420B) (arranged in descending order of their |log2(FoldChange)| values in **Table 6**). Among the G1-featured samples, except for AML870, all the other samples exhibited DNMT3A mutations. AML419A and AML328 showed FTL-3 mutations, and AML419A and AML556 showed NPM1 mutations. In contrast, the G2-featured samples did not exhibit DNMT3A, FLT3, or NPM1 mutations [**Supplementary Table S26** (22)]. These results further supported our previous comparison of mutation landscapes between G1 and G2 in the bulk RNA cohorts, indicating that DNMT3A, FLT3, and NPM1 mutations were more frequent in G1.

**Table 6:** Sample labeling based on the Scissor+ and Scissor- cell proportions.

Sample	Total cell number	Scissor+ cell number	Scissor- cell number	Scissor+ cell proportion	Scissor- cell proportion	log2(FoldChange)	Label
AML870	342	71	3	0.21	0.01	4.56	G1-featured
AML419A	1057	333	49	0.32	0.05	2.76	G1-featured
AML556	2270	716	151	0.32	0.07	2.25	G1-featured
AML328	1070	177	57	0.17	0.05	1.63	G1-featured
AML921A	3727	867	281	0.23	0.08	1.63	G1-featured
AML916	929	2	356	0.00	0.38	-7.48	G2-featured
AML707B	1507	12	636	0.01	0.42	-5.73	G2-featured
AML1012	1040	40	297	0.04	0.29	-2.89	G2-featured
AML420B	476	17	115	0.04	0.24	-2.76	G2-featured
AML329	468	95	78	0.20	0.17	0.28	Neither
AML475	372	27	33	0.07	0.09	-0.29	Neither
AML722B	78	4	5	0.05	0.06	-0.32	Neither
AML371	750	121	160	0.16	0.21	-0.40	Neither
AML210A	730	76	113	0.10	0.15	-0.57	Neither
AML314	162	2	6	0.01	0.04	-1.58	Neither
AML997	80	NA	4	NA	0.05	NA	Neither

NA, Not Available.

## Discussion

The tumor microenvironment (TME) encompasses a wide array of cell types (45), which are now recognized for their crucial roles in cancer pathogenesis. The AML TME serves a dual role. It plays a crucial role in supporting and facilitating leukemogenesis, while also exerts inhibitory effects on the proliferation of abnormal blasts, potentially impeding their progression into overt leukemia and promoting their elimination (46).

Haiment Yang et al. (41) reported significant differences in immune scores among different AML subtypes and cytogenetic risk categories, with higher immune scores correlating with poor prognosis using the TCGA-LAML transcriptome data. To investigate further, we examined immune and stromal scores in AML samples from two independent cohorts: the TCGA and Beat AML cohorts. Our results underscored the important role of immune infiltration in the AML TME. Consistent with Haiment Yang et al’s (41) results, we observed higher immune scores in AML subtypes M4 and M5 compared to other subtypes. However, in contrast to the TCGA cohort, the differences in overall survival between immune score-high and -low groups were not significant in the Beat AML cohort. Additionally, the relationship between sample immune scores and cytogenetic risk category was not consistent in the TCGA and Beat AML cohorts.

To further investigate the impact of immune conditions on AML samples at genetic and clinical levels, we focused on a set of AML-specific IRGs that effectively distinguished AML samples from normal whole blood samples. Utilizing the expression profiles of these AML-specific IRGs, we performed consensus clustering analysis, which led to the identification of two distinct immune subgroups (G1 and G2) within the TCGA cohort. Among the AML-specific IRGs, we identified key-AML-IRGs that exhibited strong associations with AML immune scores. Subsequently, we developed a subgroup prediction model incorporating the expression profiles of these key-AML-IRGs. The developed model was then applied to predict the subgroups in an independent cohort, namely the Beat AML cohort. The Scissor algorithm was employed to assign G1 or G2 features to individual AML cells in the scRNA-Seq dataset GSE116256.

Significant differences were observed between the G1 and G2 subgroups in various aspects, including clinical features, immune characterization, mutational landscapes, drug sensitivities, and putative differentiation trajectories at the single cell level.

The G1 subgroup exhibited higher immune infiltration and a more monocytic phenotype, associated with poorer prognosis, lower TIS, and higher proportions of monocytes/macrophages. In contrast, the G2 subgroup displayed lower immune infiltration and a more granulocytic phenotype, associated with better prognosis, higher TIS, and higher proportions of various immune cells. Furthermore, the expression levels of several target antigens in AML T-cell-based immunotherapy differed significantly between the G1 and G2 subgroups. This suggests that different antibody choices may be warranted in immunotherapy for samples classified into the G1 or G2 subgroups.

FLT3, DNMT3A, and NPM1 exhibited the highest mutation frequencies in G1. FLT3 and DNMT3A mutations in AML are associated with reduced survival, while the prognostic significance of NMP1 mutations depends on the presence or absence of an FLT3 mutation and its allelic ratio (47–52). Treatment options for patients with different mutation patterns of these genes vary accordingly. According to the ELN guidelines, patients with an NPM1 mutation in the absence of an FLT3 mutation falling into the favorable risk category should not undergo allogeneic hematopoietic cell transplant (HCT) at first remission due to the high risk of potentially fatal infection caused by immunosuppression and graft vs. host disease, which highlights the impact of NPM1 mutation on immune conditions in AML (52). Furthermore, mutated FLT3 (ITD) and NPM1 are leukemia-specific target antigens in AML, associated with CD8+ T-cell responses (13).

The drug sensitivity analysis in the Beat AML cohort suggested higher sensitivity of elesclomol and panobinostat in G1, while higher sensitivity of venetoclax in G2.

Elesclomol is a copper ionophore that targets mitochondrial metabolism and is being explored for cancer therapy (53–57). Our GSEA analysis in the TCGA cohort show up-regulated oxidative phosphorylation in the G1 subgroup (**Figure 4E**; **Supplementary Table S18**), suggesting a higher mitochondrial metabolic activity in this subgroup, which supported the higher sensitivity of elesclomol in G1.

Panobinostat, a histone deacetylase inhibitor (HDACi) used in treating hematological malignancies and solid tumors (58), triggers the type I interferon (IFN) pathway and promotes AML cell differentiation and therapeutic advantages (59). Jessica M. Salmon, et al. (59) suggested that up-regulated genes after panobinostat treatment were associated with type I IFN and IFN gamma (IFNγ) responses, p53 pathway, and cytokine signaling, including IL6 and TNFα in AML, which were consistent with our GSEA result (**Figures 4E, F**; **Supplementary Tables S18**, **S19**). Additionally, previous studies have shown panobinostat’s role in immune and inflammatory-related activities in antitumor processes across various cancer types (58, 60–63), corroborating our findings of a negative/positive correlation between immune scores/TIS with sample sensitivity to panobinostat (**Figures 7C, F**). Notably, panobinostat also appeared in our enrichment analysis of the top 10 differentially affected drugs/compounds between the two subgroups (**Supplementary Table S17**).

Venetoclax, an FDA-approved BCL2 inhibitor, has demonstrated efficacy in improving clinical outcomes for AML patients (64). Our findings indicated that the G1 subgroup, which had a higher proportion of M5 subtype samples and FLT3 mutation frequency, exhibited lower BCL2 expression, higher MCL1 expression, and greater resistance to venetoclax compared to the G2 subgroup. Earlier studies have also shown that M5 AML cases are more likely to be refractory to venetoclax treatment compared to non-M5 cases (65). Patients with monocytic AML have a median overall survival (OS) of 3.0 months, contrasting with 17.3 months for non-M5 AML subtypes. Furthermore, the enhanced expression of MCL1 in FLT3-ITD AML cells makes them more dependent on MCL1 for survival than on BCL2 during venetoclax treatment, leading to their survival advantage in this therapeutic context (66, 67).

By integrating analyses with the scRNA-Seq dataset GSE116256, our findings and inferences were not only reinforced but also expanded to the single-cell level. The more differentiated malignant G1-featured cells were predominantly monocyte-like cells, which characterized by elevated MCL1 expression levels, could be one of the reasons explaining the resistance of G1 subgrouped samples to venetoclax in the bulk cohorts. Notably, normal plasma cells and late-stage erythroid cells represented G2 characteristics, as indicated by their consistent labeling as Scissor- (or BC). It has been reported that in breast tumors, higher plasma cell (PC) infiltration in biopsy specimens before neoadjuvant chemotherapy was associated with pathological complete response in breast tumors. Moreover, elevated PC levels exhibited a positive correlation with favorable outcomes in patients with hormone receptor-negative breast cancer (68). Within our investigation, normal PCs were linked to G2, which was characterized by improved prognostic indicators and heightened immune activation, suggesting the potentially important role of PCs in both AML development and therapeutic strategies. In addition, myeloid neoplasms with erythroid differentiation was proved to be more resistant to venetoclax owing to their diminished reliance on the antiapoptotic protein BCL2 (69), a finding that aligns synergistically with our observations showing normal erythroid cells were G2-featured, associated with higher sensitivity to venetoclax.

While we conducted a thorough investigation into the genetic and clinical characterizations for immune-based subgroup classification, further research is essential to fully elucidate our findings and enhance their clinical relevance for risk stratification and personalized medicine. It should be noted that although RNA raw count data is ideal for differential gene expression analyses using DESeq2, we used preprocessed RSEM norm_count data from Xena to improve comparability between cohorts and mitigate batch effects (19). While this normalization addresses technical variations, it may compromise DESeq2’s ability to detect differential genes.

However, our primary objective was not to identify marker genes distinguishing AML samples from normal counterparts, but to clarify the relationship between the TME and the genetic/clinical characteristics of AML. By incorporating three independent AML cohorts, we validated our findings from both genetic and clinical perspectives at sample and cellular levels. The consistent, strongly correlated results across these diverse cohorts reinforce our conclusions. Therefore, despite the study’s limitations, we believe our findings provide a solid foundation for future investigations. Future work should involve multimodal analyses combined with laboratory examinations and clinical trials to validate and extend our findings. Evaluating the efficacy of alternative treatment decisions, such as selecting target antibodies in immunotherapy or drugs/compounds in chemotherapy, on patients with different immune conditions (classified into different subgroups) requires careful *in vitro* and *in vivo* assessment.

In summary, our study provided valuable insights into the immune landscape within AML TME. We successfully established immune-based molecular subgroups that not only encompass the clinically and genetically defined AML entities but also enhance the current prognostic classification systems. This framework holds great promise for better understanding the immune status and the associations between genotype, phenotype, and cellular hierarchies in AML, ultimately guiding informed decisions for AML therapy.

## Data Availability

The original contributions presented in the study are included in the article/**Supplementary Material**. Further inquiries can be directed to the corresponding author.
