# Supplementary material for: Immune-based subgroups uncover diverse tumor immunogenicity and implications for prognosis and precision therapy in acute myeloid leukemia
Source: Front Immunol. 2024 Nov 8;15:1451486. doi: 10.3389/fimmu.2024.1451486 (PMC11581856; doi:10.3389/fimmu.2024.1451486)
Supplement: Supplementary file 2 [file DataSheet2.docx]

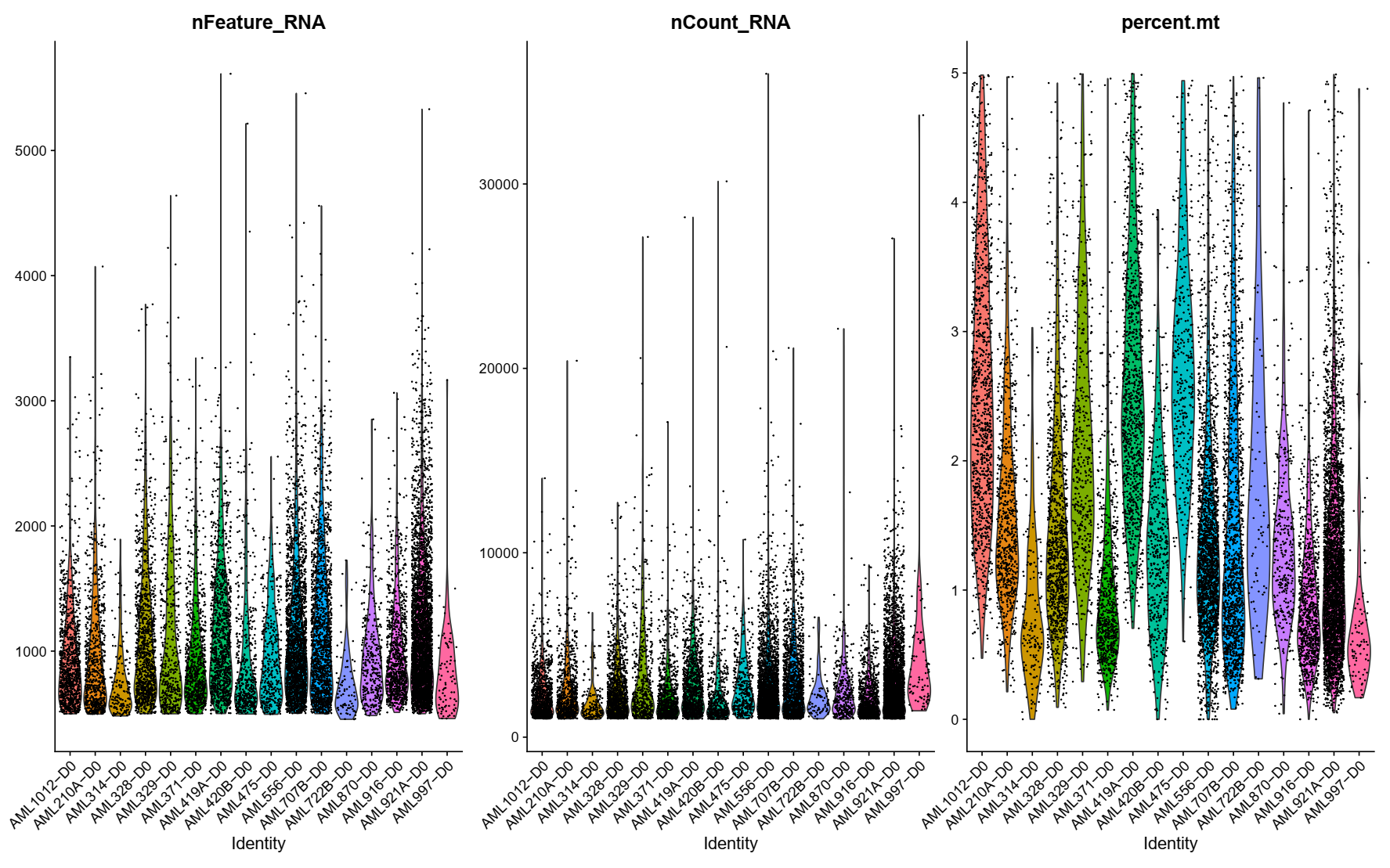


Supplementary Fig. S1. The single-cell sequencing data of 16 de novo AML samples from GSE116256.


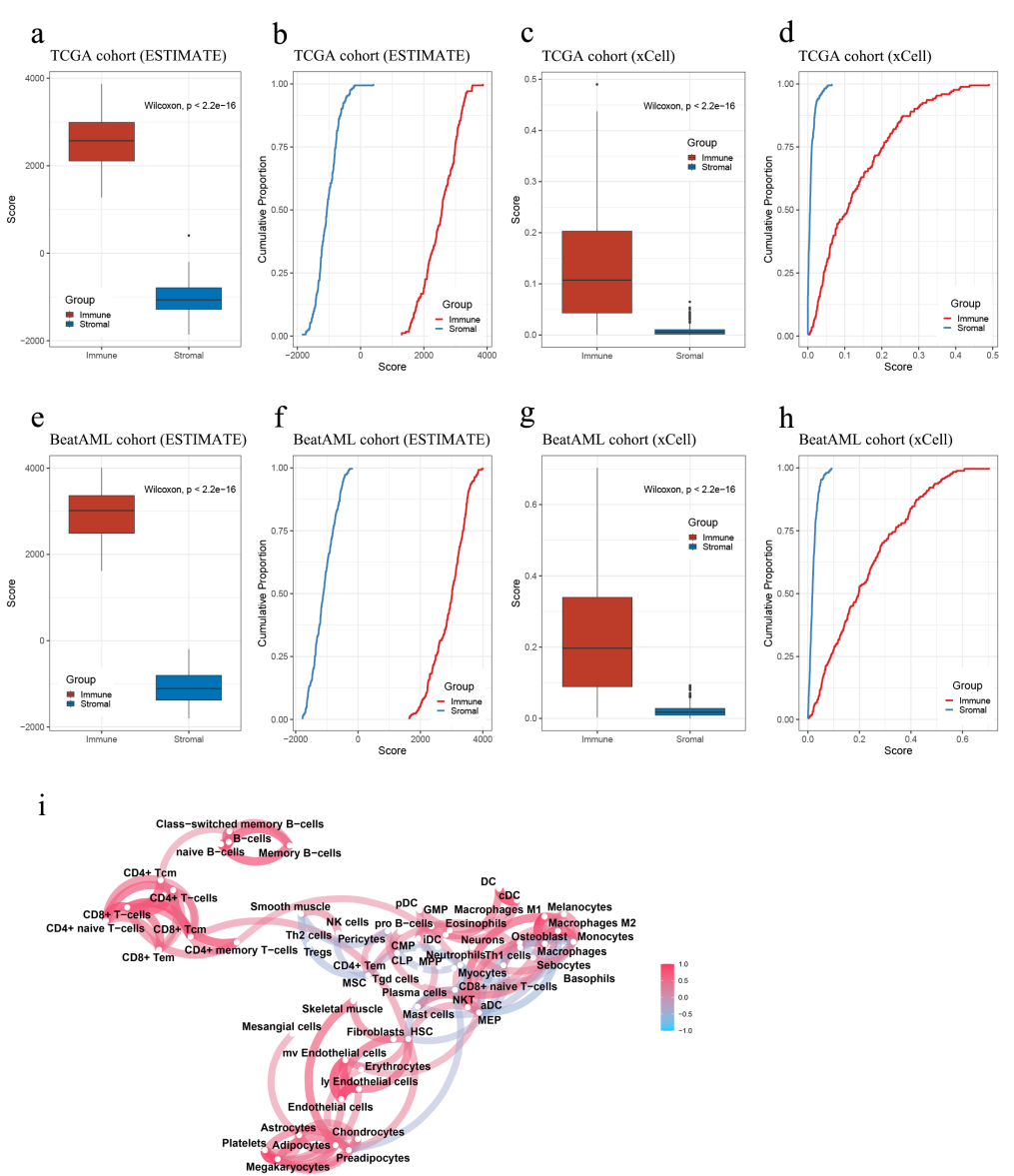


Supplementary Fig S2. The analyses of immune infiltration in the TCGA and Beat AML cohorts. a-h) Box plots and cumulative proportion curves indicated higher immune scores than stromal scores in both the TCGA and Beat AML cohorts. Immune scores consistently skewed towards significantly higher values compared to stromal scores in ESTIMATE outputs (a, b, e, f) and xCELL outputs (c, d, g, h), underscoring immune infiltration's predominant role in the AML TME; i) The correlation network depicts the intricate interconnections among diverse immune cell types within the AML TME. TME: tumor microenvironment.


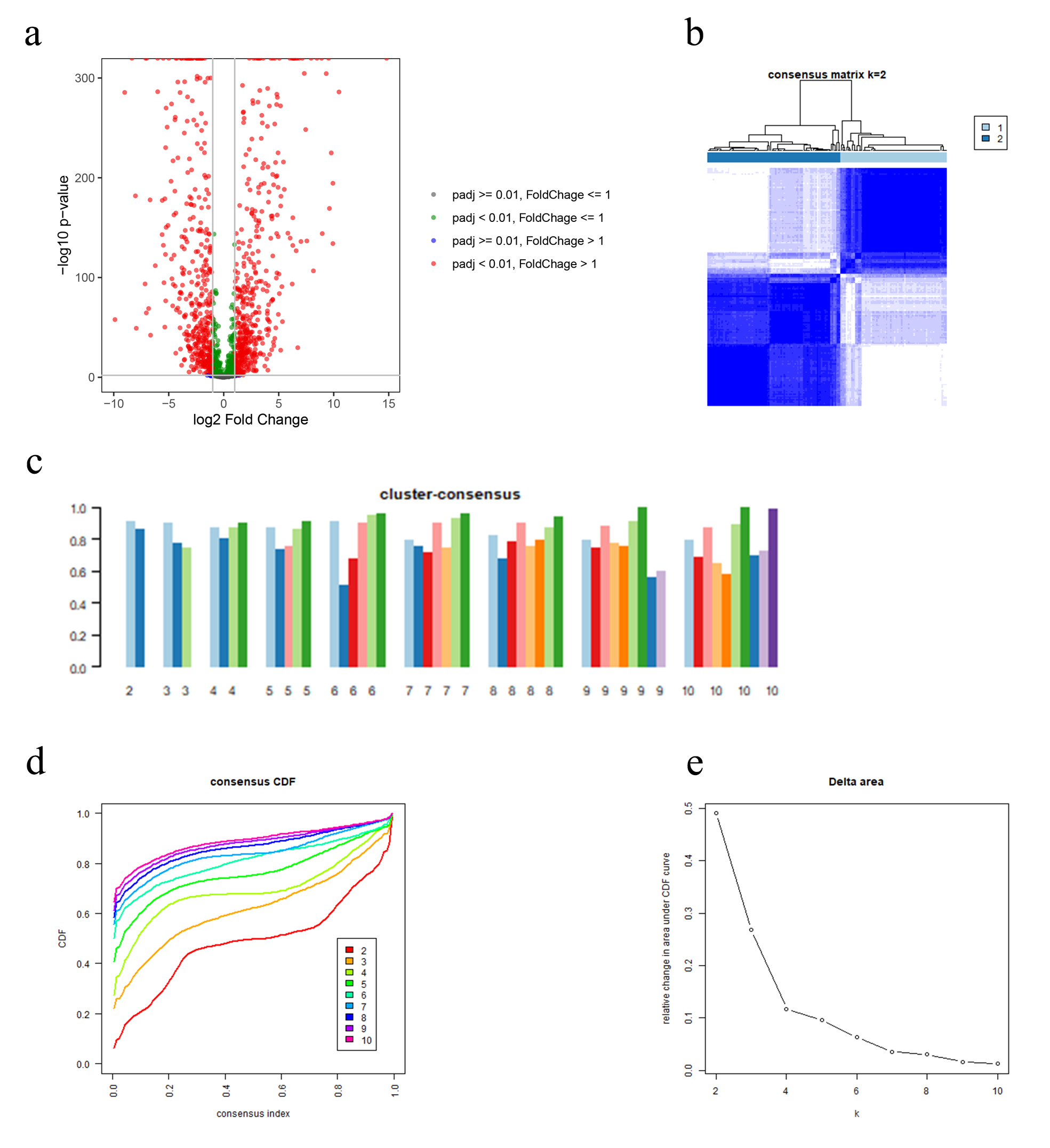


Supplementary Fig. S3. a) DESeq2 algorithm was used to identify a total of 911 AML specific IRGs between AML and normal WB samples with the filtering of padj < 0.01 and |log2(Foldchange)| > 1; b) Consensus clustering based on the expression profiling of 850 AML-specific IRGs in the TCGA cohort (n = 173); c) Consensus scores for each subtype when k=2 to 10; d) CDF plot of consensus clustering, illustrating the CDF curve as the number of clusters varies; e) Delta Area plot, depicting the relative change in the AUC of the CDF as the number of clusters increases. IRG: immune-related gene; CDF: cumulative distribution function; AUC: area under the curve.


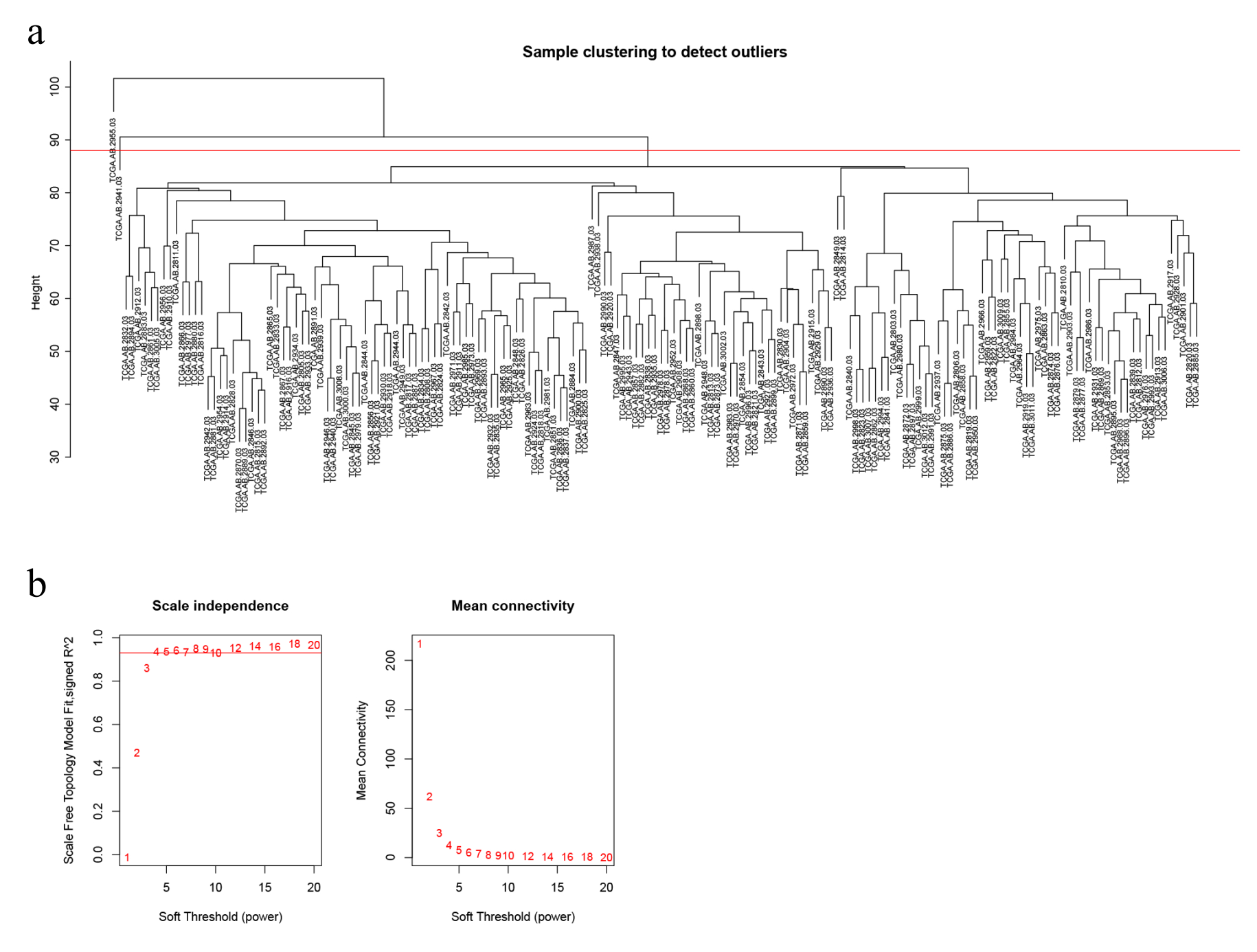


**Supplementary Fig. S4. Using WGCNA to identify AML-immune-specific modules and key-AML-IRGs.** a) Outlier samples removing for WGCNA analysis. The red line indicated a branch cut; b) Analysis of network topology for various soft-thresholding powers. Here, we selected 4 as the power of the β value.


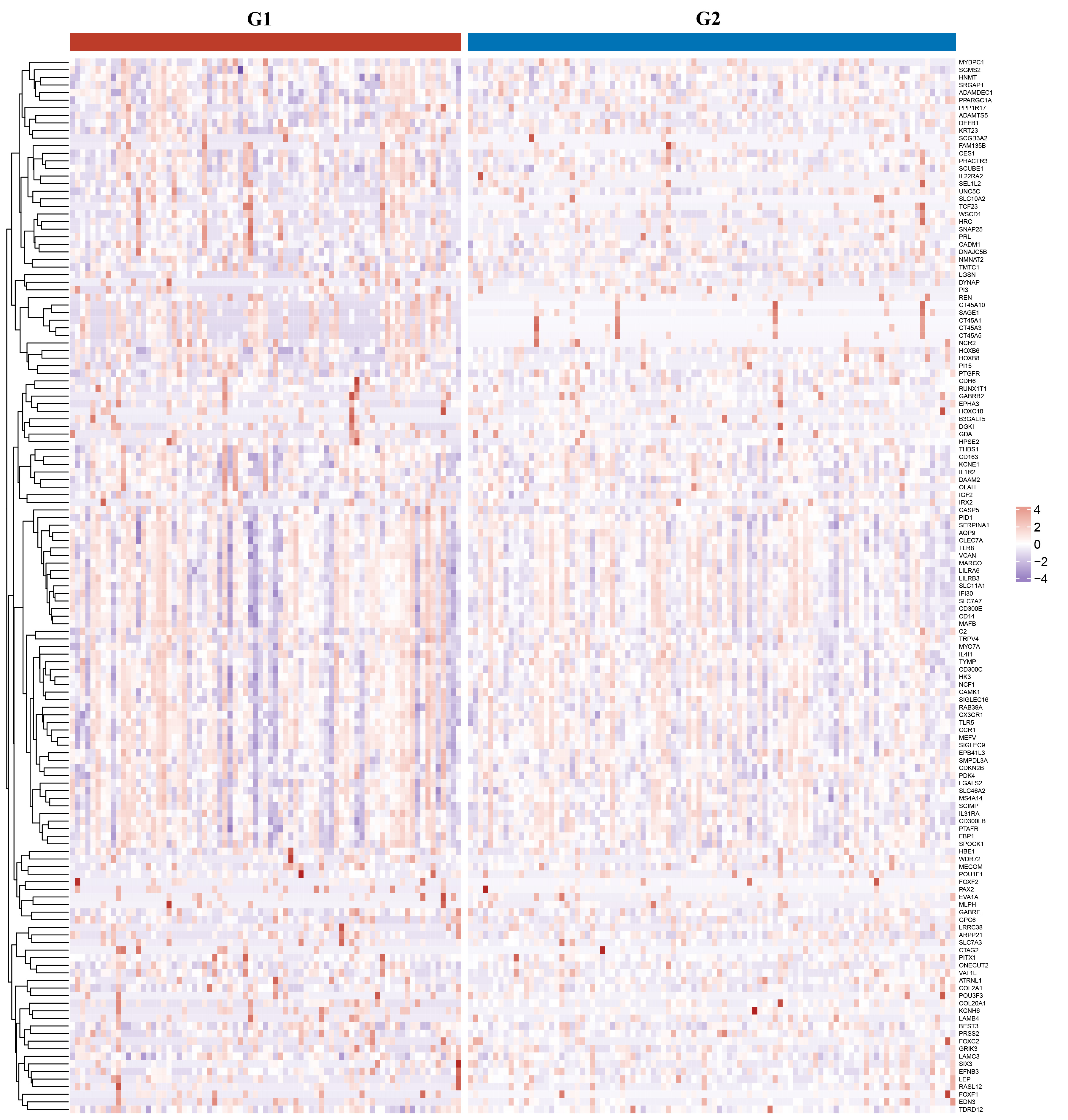


**Supplementary Fig. S5.** **Heatmap of Robust DEGs in the TCGA cohort between G1 and G2 subgroups.** The figure displays a total of 139 genes, highlighting the differential expression patterns between the two subgroups.


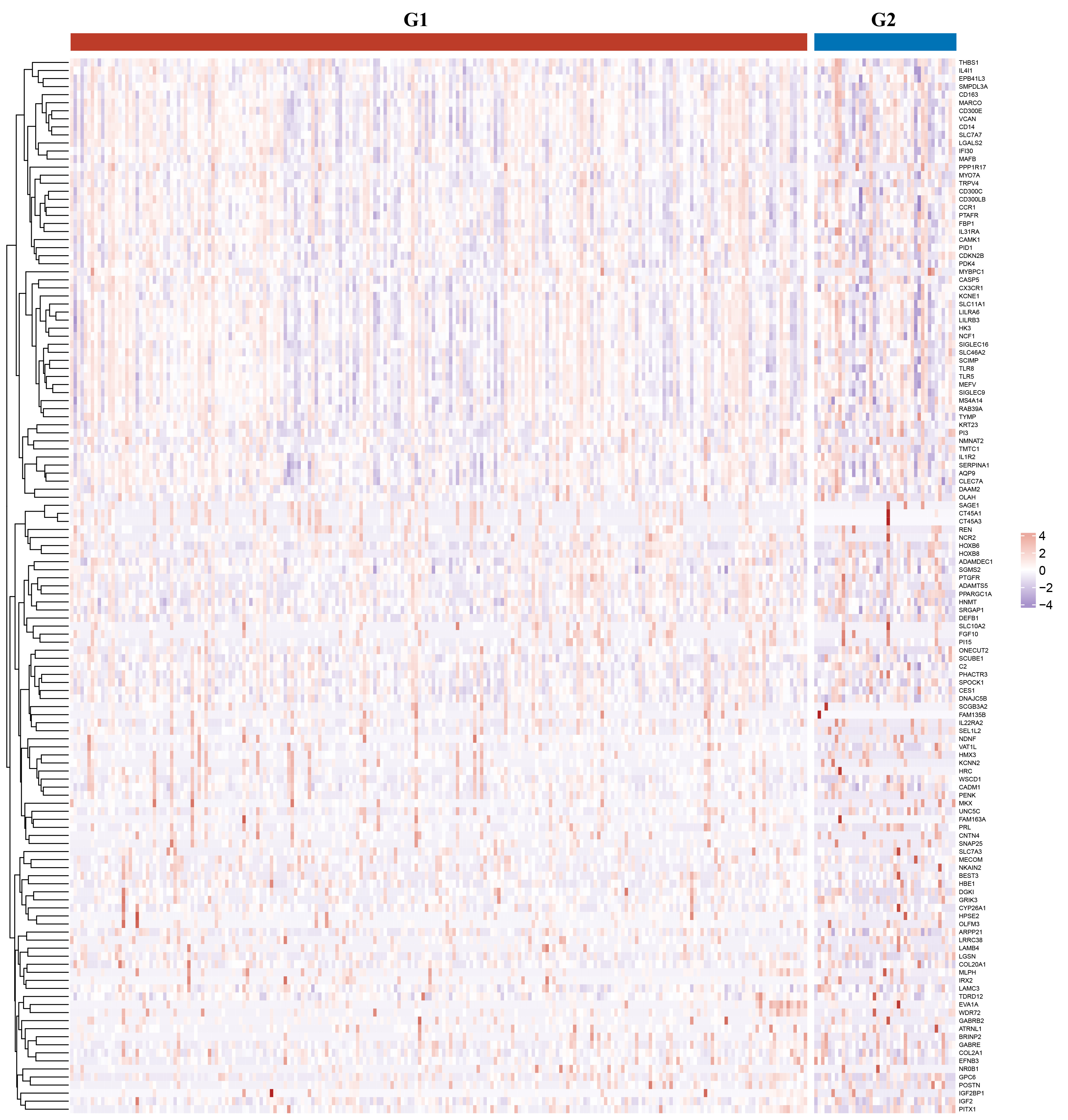


**Supplementary Fig. S6.** **Heatmap of Robust DEGs in the Beat AML cohort between G1 and G2 subgroups.** The figure displays a total of 131 genes, highlighting the differential expression patterns between the two subgroups.
